# A comprehensive *in-vitro*/*in-vivo* screening toolbox for the elucidation of glucose homeostasis modulating properties of plant extracts (from roots) and its bioactives

**DOI:** 10.3389/fphar.2024.1396292

**Published:** 2024-06-26

**Authors:** Ilka Bauer, Gerald Rimbach, Sönke Cordeiro, Anja Bosy-Westphal, Julian Weghuber, Ignacio R. Ipharraguerre, Kai Lüersen

**Affiliations:** ^1^ Division of Food Sciences, Institute of Human Nutrition and Food Science, University of Kiel, Kiel, Germany; ^2^ Institute of Physiology, University of Kiel, Kiel, Germany; ^3^ Division of Human Nutrition, Institute of Human Nutrition and Food Science, University of Kiel, Kiel, Germany; ^4^ Center of Excellence Food Technology and Nutrition, University of Applied Sciences Upper Austria, Wels, Austria; ^5^ FFoQSI—Austrian Competence Centre for Feed and Food Quality, Safety & Innovation, Tulln, Austria

**Keywords:** carbohydrate-hydrolysing enzymes, ussing chamber, sodium-dependent glucose transporter 1, dipeptidyl-peptidase 4, glucose transporter 4, KATP channel, *Drosophila melanogaster*, hen’s egg test

## Abstract

Plant extracts are increasingly recognized for their potential in modulating (postprandial) blood glucose levels. In this context, root extracts are of particular interest due to their high concentrations and often unique spectrum of plant bioactives. To identify new plant species with potential glucose-lowering activity, simple and robust methodologies are often required. For this narrative review, literature was sourced from scientific databases (primarily PubMed) in the period from June 2022 to January 2024. The regulatory targets of glucose homeostasis that could be modulated by bioactive plant compounds were used as search terms, either alone or in combination with the keyword “root extract”. As a result, we present a comprehensive methodological toolbox for studying the glucose homeostasis modulating properties of plant extracts and its constituents. The described assays encompass *in-vitro* investigations involving enzyme inhibition (α-amylase, α-glucosidase, dipeptidyl peptidase 4), assessment of sodium-dependent glucose transporter 1 activity, and evaluation of glucose transporter 4 translocation. Furthermore, we describe a patch-clamp technique to assess the impact of extracts on K_ATP_ channels. While validating *in-vitro* findings in living organisms is imperative, we introduce two screenable *in-vivo* models (the hen’s egg test and *Drosophila melanogaster*). Given that evaluation of the bioactivity of plant extracts in rodents and humans represents the current gold standard, we include approaches addressing this aspect. In summary, this review offers a systematic guide for screening plant extracts regarding their influence on key regulatory elements of glucose homeostasis, culminating in the assessment of their potential efficacy *in-vivo*. Moreover, application of the presented toolbox might contribute to further close the knowledge gap on the precise mechanisms of action of plant-derived compounds.

## 1 Introduction

Insufficient reduction of (postprandial) blood glucose levels is strongly associated with the pathogenesis of obesity and chronic metabolic disorders such as type 2 diabetes mellitus and cardiovascular diseases (Blaak et al., 2012). High postprandial glucose levels are known to cause microvascular dysfunction (Aroda and Eckel, 2022), and are also a prevailing risk factor for cardiovascular mortality even in the healthy population (European Diabetes Epidemiology Group, 1999). This may in part be due to the enhanced postprandial insulin levels in response to impaired glucose tolerance (Pyörälä et al., 1998; Shen et al., 2022), emphasising the importance of preventing increased postprandial glucose levels and subsequent progressive development of prediabetic phenotypes. Interestingly, lowering postprandial glucose peaks seems to be the more relevant target than lowering average blood glucose levels/HbA1c with regard to cardiovascular disease prevention (Hanssen et al., 2020).

**FIGURE 1:**
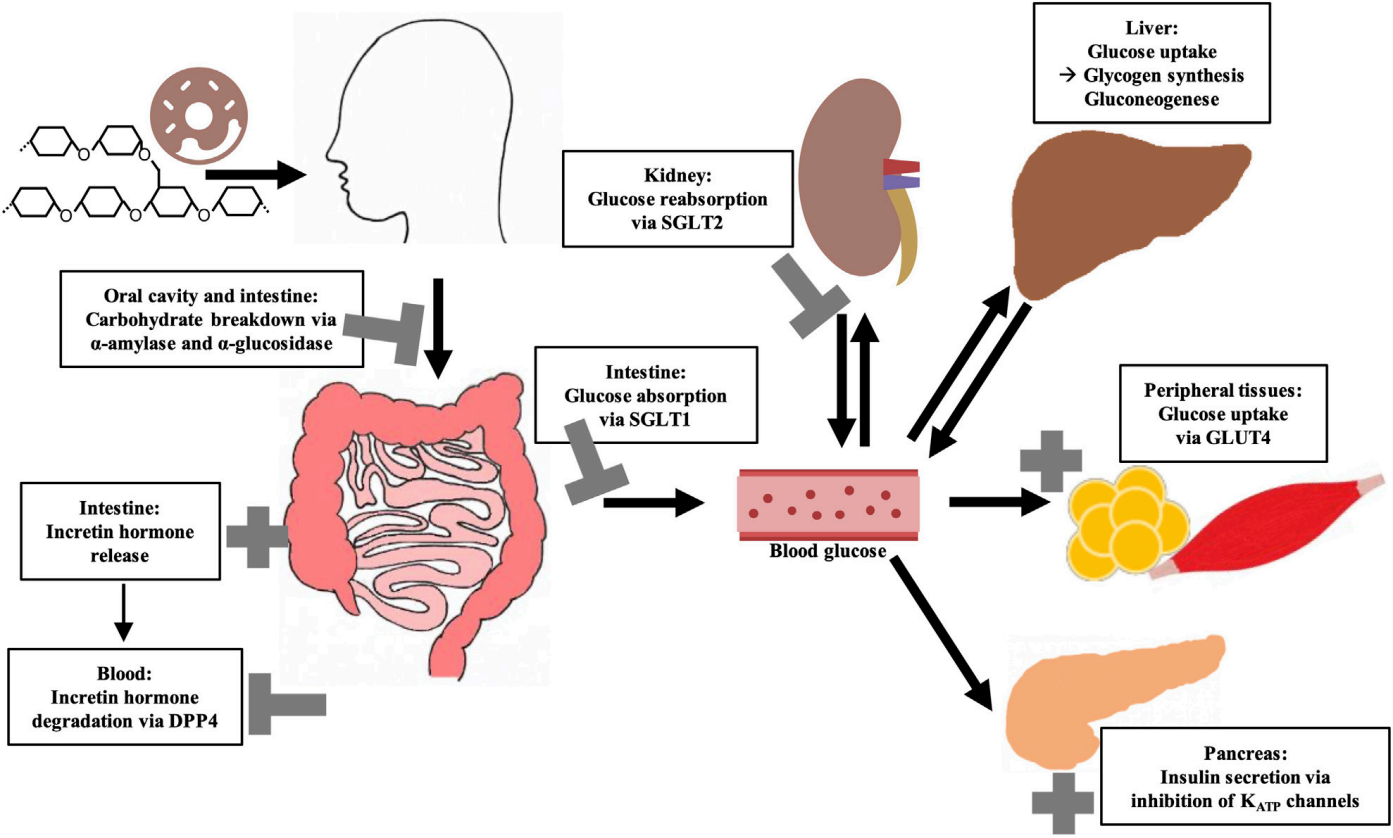
Overview of key regulatory elements of (postprandial) blood glucose regulation and their possible modulation by bioactive compounds from plant extracts. Sodium-dependent glucose transporter (SGLT); Glucose transporter 4 (GLUT4); Dipeptidyl peptidase 4 (DPP4).

The blood glucose concentration is dependent on the postprandial absorption rate of glucose, the endogenous production of glucose, the uptake of glucose by the liver and peripheral tissues as well as the disposal of glucose (Wu et al., 2020). Among these, gastrointestinal responses to meal ingestion (i.e., gastric emptying, intestinal digestion and absorption, and the secretion of gastrointestinal hormones) are key determinants of postprandial glucose levels. In order to improve glycemic control, different strategies can be employed. These comprise lifestyle interventions such as the modulation of physical activity and diet, but also the prescription of (oral) hypoglycemic medications (Pasmans et al., 2022). Plant-based medications which are assumed to have benefits in availability, side effects, and financial issues over conventional (oral) antihyperglycemic drugs (Salehi et al., 2019), might be a promising and effective strategy as well. In this context, it should be emphasized that plant extracts are a complex mixture of putative bioactive compounds, that have the potential to act at multiple targets simultaneously, including various enzymes or transporters involved in the regulation of glucose homeostasis. Figure 1 summarizes the most important mechanisms by which plant extracts or plant-derived molecules may affect postprandial blood glucose levels.

**FIGURE 2:**
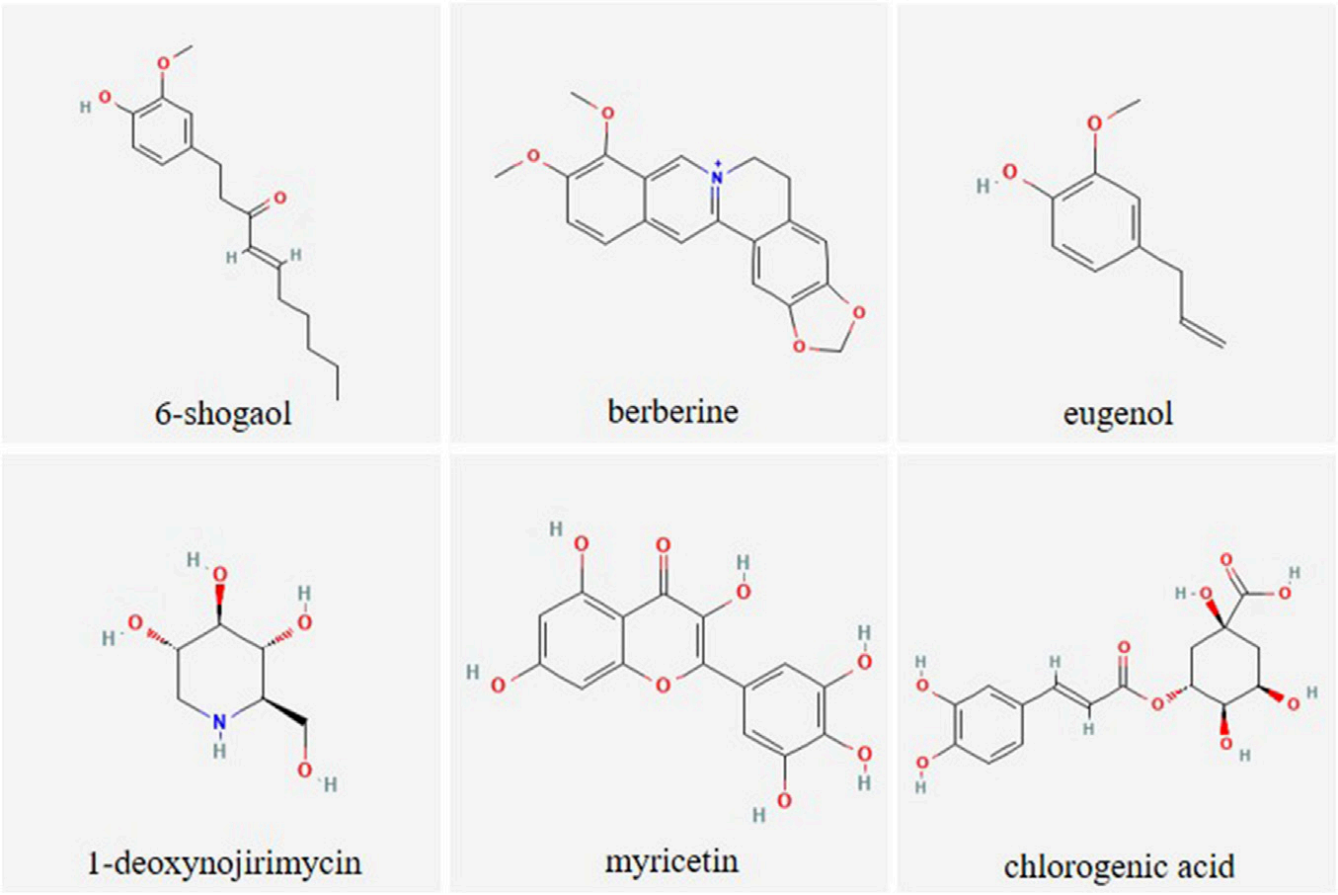
Chemical structure of selected plant-derived compounds with putative antihyperglycemic activity. The structures of 6-shogaol (PubChem CID: 5281794), berberine (PubChem CID: 2353), eugenol (PubChem CID: 3314), 1-deoxynojirimycin (PubChem CID: 29435), myricetin (PubChem CID: 5281672) and chlorogenic acid (PubChem CID: 1794427) were taken from PubChem (Kim et al., 2023)

Among the most frequently studied putative antihyperglycemic plant species are *Morus alba* L., *Cinnamomum zeylanicum* J. Presl, *Trigonella foenum-graecum* L., *Phaseolus vulgaris* L., *Zingiber officinale* Rosc., and *Panax ginseng* C.A.Meyer as recently summarized. On the one hand, this is due to the fact that the bioactive lead substances are identified here, such as phaseolamine from *P. vulgaris*, ginsenosides from *P. ginseng* or shogaol and gingerol from *Z. officinale*. On the other hand, these plant species are readily available, low cost and generally considered safe (Przeor, 2022). Interestingly, a combined formulation of *M. alba*, *P. vulgaris* and green coffee extract showed high potential to lower postprandial blood glucose and insulin concentrations in randomized, double-blind, placebo-controlled, cross-over experiments, which is attributed to the impact of bioactive compounds in the extracts on different regulatory pathways of glucose homeostasis (Adamska-Patruno et al., 2018). Figure 2 gives the structure of selected plant-derived compounds that are highly recognized for their putative antihyperglycemic effects, namely, 6-shogaol (Yi et al., 2019), berberine (Utami et al., 2023), eugenol (Srinivasan et al., 2014), 1-deoxynojirimycin (Ramappa et al., 2020), myricetin (Imran et al., 2021), and chlorogenic acid (Zuñiga et al., 2018).

However, there is a great effort in research to discover novel antihyperglycemic plants and their related bioactive compounds and to decipher their mechanisms of action, with the overall intention to identify even more potent plant-based compounds or combination of these. Collections of plant extracts, commonly established by biotechnology companies and academic research institutes, are a tremendous help when a large number of plant extracts should be screened (Onur et al., 2013; Potterat and Hamburger, 2014; Wilson et al., 2020). For example, the screening library established by the US National Cancer Institute is one of the most relevant with over 230,000 extracts (McCloud, 2010; Thornburg et al., 2018). The largest French extract library of the Institut de Chimie des Substances Naturelles contains approximately 14,000 extracts (Marinetti, 2018). Extracts from 4,000 different plant species can be purchased from the company PhytoPharmacon (PhytoPharmacon, n. d.). The German “Plant Extract Collection Kiel in Schleswig-Holstein” (PECKISH) is a screening library with over 4,500 extracts, which is distinguished by its open accessibility (Onur et al., 2013). In this context, well documented, extensive plant extract libraries can be used to generate tailored sub-libraries selecting extracts with specific properties, e.g., extracts based on plants from the same region, plant family or plant part. In this regard, the targeted use of plant root extracts derived from such libraries might be of particular interest. In general, the extensive use of plant roots and rhizomes is frequently described in the traditional medicine of various cultures worldwide (Bais et al., 2001), as for example, by traditional healers (called “root doctors”) in Angola (Novotna et al., 2020). Despite this, as indicated by reviews concerning antihyperglycemic activity of medicinal plant extracts, underground plant material is not as widely studied compared to the aerial parts (Bailey and Day, 1989; Arumugam et al., 2013; Mamun-or-Rashid et al., 2014; Ardalani et al., 2021; Kifle et al., 2022). At this point, it should be noted that the occurrence and concentration of bioactive compounds within a plant species can vary greatly depending on the plant part. For example, leaves apparently have the highest content of flavonoids (Hao et al., 2023) although these are also present in the roots. However, additional bioactive compounds, such as phenols, alkaloids, phytosteroids, saponins, tannins, terpenoids, anthraquinones, and cardiac glycosides, which are abundant in roots, make these plant parts particularly interesting (Ardalani et al., 2021). Moreover, roots are one of the most important plant organs for accumulating these active components (Li et al., 2020). A root-specific metabolism leads to uniquely synthesized compounds in the root system, which are generally considered to have great potential for pharmacological applications, such as forskolin (diterpenoid) or the shikonins (group of naphthoquinones) (Bais et al., 2001; Ardalani et al., 2021).

However, easy-to-use methods are crucial enabling plant (root derived) extracts to be screened for multiple targets. In addition, alternative *in-vivo* models capable of replacing classical rodent studies, while being without ethical concerns and less time-consuming, are becoming increasingly important.

In the first part of this review, major regulatory elements of glucose homeostasis that might be modulated by plant extracts are outlined. The currently used antihyperglycemic drugs are considered in context of these molecular targets. In the second part, a comprehensive methodological toolbox including *in-vitro* and *in-vivo* assays related to the regulatory elements is presented that enables screening and verification of plant (root) extracts and bioactive compounds with respect to their antihyperglycemic properties. The assays described herein cover well known standard methods (e.g., spectrophotometric determination of α-amylase and α-glucosidase activity) but also less common methods such as patch-clamp measurements for K_ATP_ channels. Likewise, the *in-vivo* assays presented, using hen’s eggs and *Drosophila melanogaster* (*D. melanogaster*) as model organisms, are not yet widely used in the context of blood glucose regulation. Advantages and limitations of the different methods are discussed. In particular, this extensive collection of assays could facilitate the systematic investigation of novel plant material for future research and further decipher their mechanisms of action. Finally, root extracts (which could be particularly interesting for research in the future, but have not yet received widespread attention) targeting the aforementioned glucose homeostasis pathways are summarized in particular.

## 2 Key regulatory elements of glucose homeostasis

### 2.1 Dietary carbohydrate digestion

**FIGURE 3:**
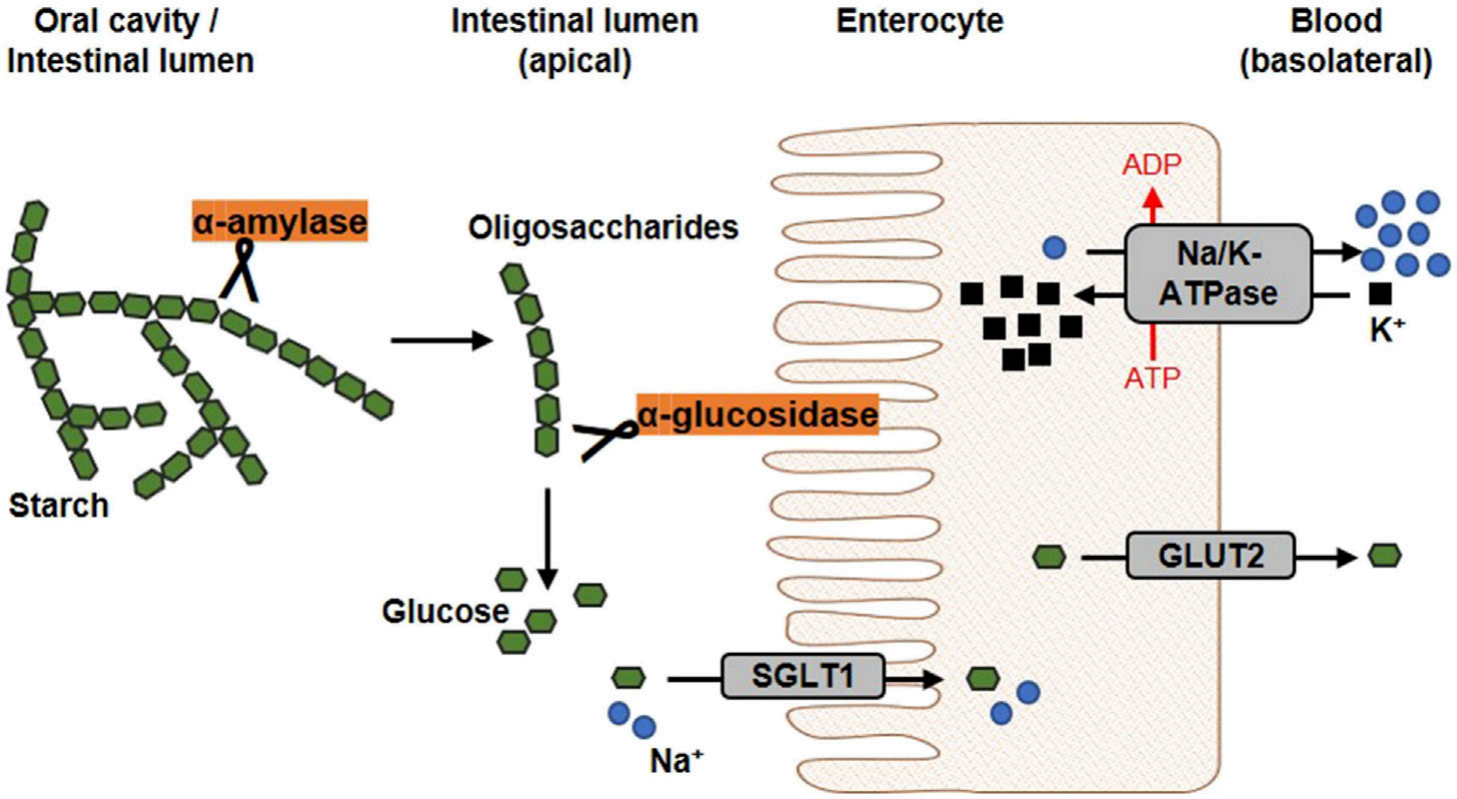
Schematic overview of glucose release and transport. In the oral cavity and the gut, the enzyme α-amylase, secreted by salivary glands and the pancreas, catalyses the hydrolysis of internal α-1,4-glycosidic bonds in dietary starch molecules. In the intestinal lumen, the resulting oligosaccharides are further broken down by the membrane-bound α-glucosidase. The α-glucosidase catalyses the hydrolysis of terminal α-glycosidic bonds thus releases glucose. At the apical membrane of enterocytes, one molecule glucose and two sodium ions are co-transported into the cell via the sodium-dependent glucose transporter 1 (SGLT1). Glucose is transported into the circulation on the basolateral side via passive diffusion through the Glucose transporter 2 (GLUT2). The driving force of the intestinal glucose uptake is a sodium gradient established under energy consumption by the Na^+^/K^+^-ATPase, which transported sodium basolaterally out of the cell (modified from (Koepsell, 2020; Barber et al., 2021). Used under CC BY 4.0: https://creativecommons.org/licenses/by/4.0/.

The diet usually contains a mixture of mono-, di- and polysaccharides such as glucose, sucrose and starch. Within the digestive tract, complex carbohydrates are initially converted into monosaccharides before they are absorbed and lead to a postprandial elevation of blood glucose level. The first step of dietary starch breakdown is accomplished by the enzyme α-amylase, which is predominantly secreted by salivary glands and the pancreas (Zakowski and Bruns, 1985). As a member of endoenzymes, α-amylase degrades complex starch molecules into oligosaccharides of 6 - 8 glucose units by catalysing the hydrolysis of internal α-1,4 glycosidic bonds (Sales et al., 2012). Since neither terminal glucose groups nor α-1,6 glycosidic bonds can be cleaved by this enzyme (Whitcomb and Lowe, 2007), both linear and branched products are generated that have to be broken down by further degrading enzymes. α-Glucosidases, located on the brush border membrane of the small intestine, catalyses the hydrolysis of α-1,4 and α-1,6 glycosidic bonds of α-amylase degradation products and also of dietary disaccharides via an exo-mechanism thereby releasing α-glucose from the non-reducing end of the substrate which can be absorbed by enterocytes (Chiba, 1997) (Figure 3). Interestingly, about 80% of starch can be broken down into glucose by α-glucosidase without the involvement of α-amylase (Lin et al., 2010; Lin et al., 2012). As these, enzyme actions, which can be considered as a rate-limiting step in carbohydrate digestion, are relatively easy to access pharmacologically due to their location within the gastrointestinal tract, both α-amylase and α-glucosidase are favoured targets of antihyperglycemic medications.

Acarbose, a pseudo-oligosaccharide of microbial origin derived from cultures of the soil bacteria *Actinoplanes* sp., is a well-known α-amylase and α-glucosidase inhibitor (Wehmeier and Piepersberg, 2004). Acarbose is composed of an unsaturated cyclitol, an amino-deoxyhexose and a maltose moiety and is recognized as a substrate by hydrolase enzymes involved in di- and polysaccharide digestion (Wehmeier, 2003; Tsunoda et al., 2022). However, the nitrogen linkage between the cyclitol (non-reducing end) and the deoxyhexose cannot be cleaved by these enzymes (Li et al., 2005; Tsunoda et al., 2022). Moreover, acarbose has an increased binding affinity for α-glucosidase compared to dietary saccharides (Rosak and Mertes, 2012). Acarbose is reported as a competitive inhibitor for α-glucosidase and a mixed non-competitive inhibitor for α-amylase (Kim et al., 1999). Other α-glucosidase inhibitors to be named are miglitol and voglibose. However, all these inhibitors are limited by their relatively high production cost, for example, due to complex biosynthesis for acarbose (Zhao Q. et al., 2017), and gastrointestinal (GI) adverse effects such as flatulence, abdominal discomfort and diarrhea caused by fermentation of carbohydrates by gut bacteria (Krentz and Bailey, 2005).

### 2.2 Glucose absorption through sodium-dependent glucose transporters

Cellular uptake of glucose from diet or blood is mediated by integral transport proteins. Depending on the transport mechanism, glucose transporters are classified into two families: (i) the facilitative glucose transporters (GLUTs) using a diffusion gradient and (ii) the sodium-dependent glucose co-transporters (SGLTs) through which glucose is actively transported by a Na^+^-electrochemical gradient (Bell et al., 1990; Wood and Trayhurn, 2003). Various isoforms of GLUTs and SGLTs have been identified showing different tissue distribution patterns (Wright et al., 2011; Mueckler and Thorens, 2013). The predominant glucose transporters in the intestine enabling dietary glucose (and galactose) uptake into circulation are SGLT1 and GLUT2 (Gorboulev et al., 2012). In Figure 3, the generally accepted mechanism of intestinal glucose absorption is depicted. SGLT1 is localized in the brush-border membrane of enterocytes, with its expression upregulated by dietary sugar (Margolskee et al., 2007; Song et al., 2016). Along with sodium, glucose is taken up into epithelial cells via SGLT1, whereas GLUT2 mediates its release into circulation on the basolateral side following the concentration gradient. Apparently, suppression of the SGLT1 transport protein can attenuate postprandial glucose peaks.

Much attention is currently also being given to another isoform of the SGLT family, namely, SGLT2 (Song et al., 2016). This low-affinity, high-capacity glucose transporter accounts for approximately 90% of renal glucose reabsorption, while the remaining 10% are reabsorbed by the high-affinity, low-capacity transporter SGLT1 (Kanai et al., 1994). SGLT2 inhibitors (e.g., gliflozins) have the potential not only to improve glycemic control by increasing glucose excretion in the urine (Chao and Henry, 2010; Scheen, 2015), but are also reported to have cardiovascular benefits (Zelniker et al., 2019). However, even if SGLT2 is completely blocked, glucose reabsorption in the kidney is partially maintained by SGLT1, indicating the prospective potential of dual SGLT1/2 inhibitors (Powell et al., 2013).

Phlorizin, a glycoside of the dihydrochalcone phloretin, was first isolated from the bark of the apple tree in 1835. This natural compound turned out to be the first non-selective inhibitor of both SGLT isoforms with a competitive mode of action (Ehrenkranz et al., 2005). However, phlorizin entails disadvantages e.g., poor water solubility, low oral bioavailability (Crespy et al., 2001) and rapid degradation (Tian et al., 2021), but the structure of phlorizin has been served as a template for (synthetic) SGLT inhibitors, which comprise individual SGLT2 and SGLT1 inhibitors as well as dual SGLT1/2 blockers (Dominguez Rieg and Rieg, 2019). Nevertheless, the discovery of novel natural SGLT inhibitors is becoming increasingly important (Oranje et al., 2019).

### 2.3 The incretin system

The presence of dietary carbohydrates triggers a group of intestinal hormones known as the incretin system. An important step in the discovery of the incretin system was made in 1964 when it was shown for the first time that oral glucose provided a more potent insulin response compared with intravenous glucose stimulus (Elrick et al., 1964; McIntyre et al., 1964; Rehfeld, 2018). Nowadays it is known that this phenomenon is mainly due to two gut-derived hormones: glucose-dependent insulinotropic polypeptide (GIP) and glucagon-like peptide-1 (GLP-1) (Drucker and Nauck, 2006; Rehfeld, 2018). While the plasma level of incretin hormones is low in the fasting state, it increases within minutes after meal ingestion (Drucker and Nauck, 2006). Subsequently, GIP and GLP-1 stimulate insulin secretion form the pancreas by binding to their receptors GIPR and GLP-1R (Mayendraraj et al., 2022). Thus, incretin hormones play a particularly important role in postprandial glucose regulation being responsible for approximately 70% of insulin secretion after eating (Gallwitz, 2019). Other incretin effects have also been described, such as decelerated gastric emptying or reduced appetite (Drucker and Nauck, 2006). Interestingly, modulation of the incretin effect has proven to be a remarkably effective strategy for the management of obesity, as recently demonstrated by the therapeutic use of semaglutide (Chao et al., 2023).

**FIGURE 4:**
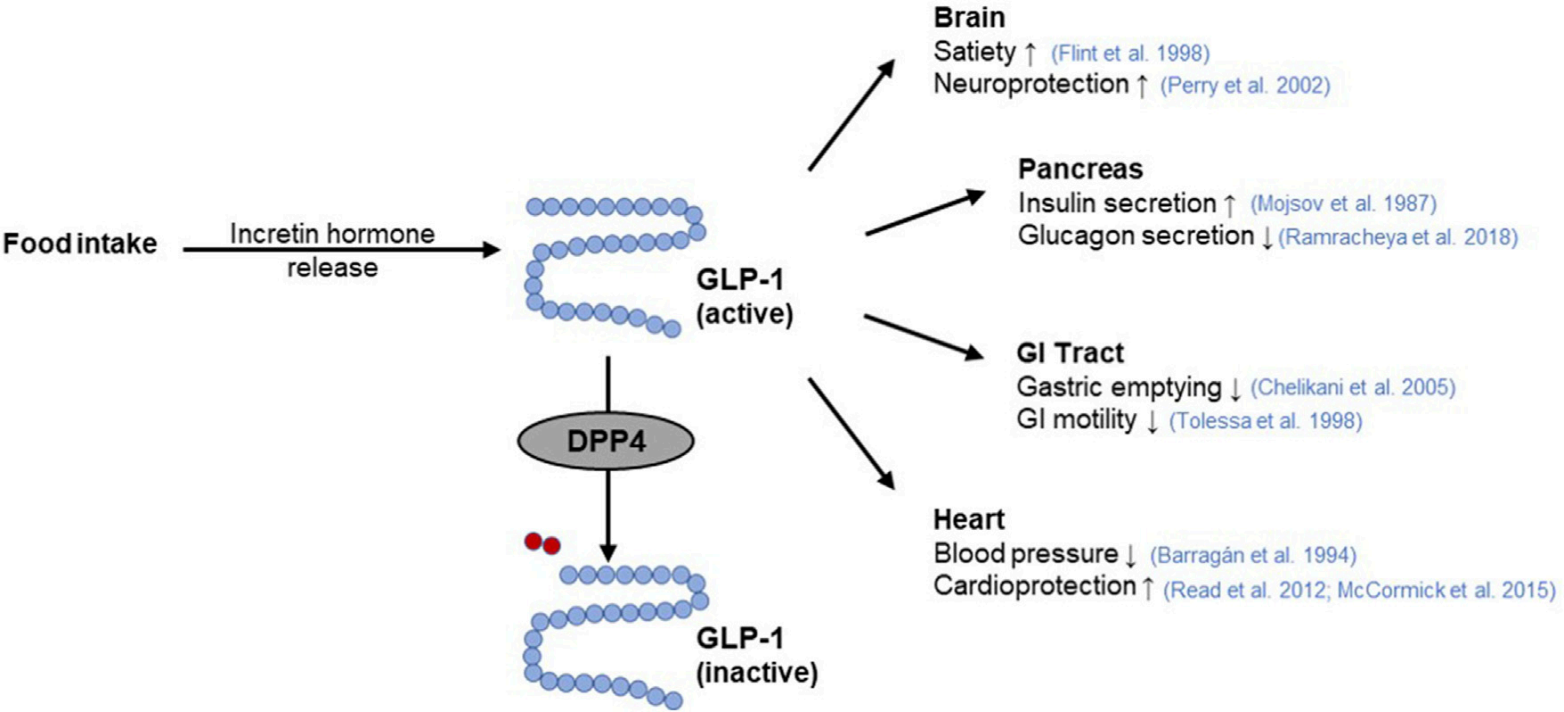
GLP-1 function and its interaction with DPP4. The incretin hormone glucagon-like peptide-1 (GLP-1) is released from intestinal cells as a response to nutrient intake. Subsequently, GLP-1 elicits pleiotropic effects on the target organs such as the pancreas (Mojsov et al., 1987; Ramracheya et al., 2018), brain (Flint et al., 1998; Perry et al., 2002), gastrointestinal (GI) tract (Tolessa et al., 1998; Chelikani et al., 2005), or heart (Barragán et al., 1994; Read et al., 2012; McCormick et al., 2015). Rapid inactivation of GLP-1 occurs by cleavage of a terminal dipeptide by the enzyme dipeptidyl peptidase 4 (DPP4) (modified from (Saraiva and Sposito, 2014; Razavi et al., 2022)). Used under CC BY 4.0: https://creativecommons.org/licenses/by/4.0/.

The incretin system represents an excellent target for the modulation of glucose homeostasis (Holst et al., 2009) via two different strategies. On the one hand, GLP-1 receptor agonists bind to the GLP-1R stimulating pancreatic insulin secretion, thereby improving postprandial glucose levels (Meier, 2012; Gilbert and Pratley, 2020). On the other hand, GIP and GLP-1 are substrates for the enzyme dipeptidyl peptidase 4 (DPP4), whose cleavage activity leads to their rapid degradation (Figure 4) (Mentlein et al., 1993). Hence, inhibitors of regulatory DPP4 can be used to prolong the circulating half-life of incretin hormones (Gilbert and Pratley, 2020).

Several GLP-1 receptor agonists e.g., exenatide, lixisenatide, liraglutide or albiglutide as well as DPP4 inhibitors, known as gliptins such as sitagliptin, vildagliptin, linagliptin, saxagliptin or allogliptin are established as a class of oral hypoglycemic medications (Meier, 2012; Gallwitz, 2019; Gilbert and Pratley, 2020). Since there is evidence that SGLT1 inhibition increases plasma GLP-1 concentrations (Powell et al., 2013; Oguma et al., 2015; Io et al., 2019), combined use of SGLT1 and DPP4 inhibitors may be a particularly potent strategy for improved glycemic control (Zambrowicz et al., 2013). However, both GLP-1 receptor agonists and DPP4 inhibitors are associated with adverse effects. While DPP4 inhibitors are mainly associated with headache, nasal pharyngitis, and upper respiratory tract infections, GLP-1 receptor agonists are reported to contribute to gastrointestinal complaints (Gilbert and Pratley, 2020).

### 2.4 Pancreatic β-cell K_ATP_ channel activity

**FIGURE 5:**
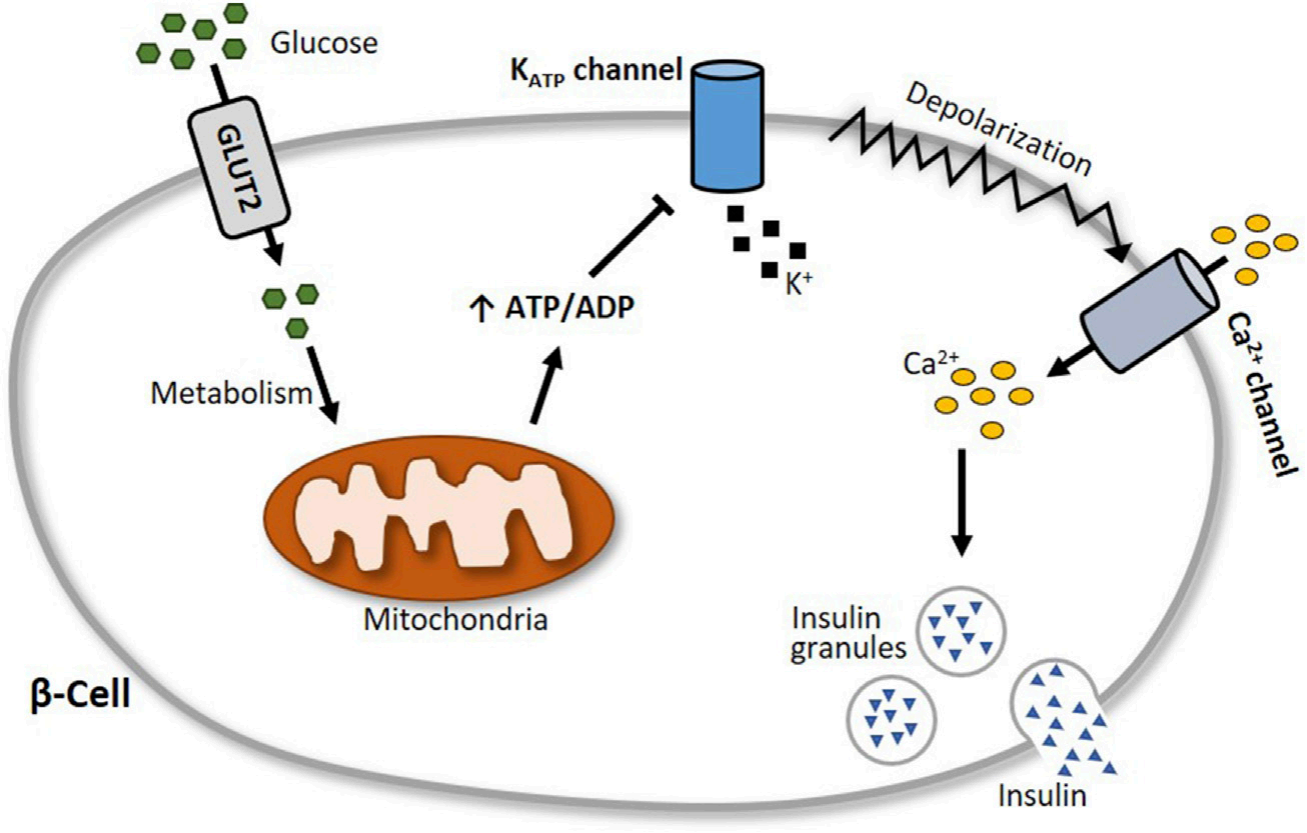
Schematic illustration of glucose-stimulated insulin secretion. At elevated blood glucose levels, more glucose is transported via glucose transporter 2 (GLUT2) into pancreatic β-cells. Subsequent glucose metabolism results in an augmented generation of ATP, which closes the ATP-sensitive K_ATP_ channel Kir6.2. This leads to a depolarization of the cell membrane and an opening of voltage-gated Ca^2+^ channels, followed by an influx of calcium into the cell. The increase of intracellular Ca^2+^ levels then triggers the exocytosis of insulin granules.

Insulin is considered the major blood glucose-lowering hormone. It is produced and stored in the β-cells of the pancreas until its release, which is in part triggered by glucose entry into the cells and subsequent alterations in the activity of ion channels (Ashcroft et al., 1984; Ashcroft and Rorsman, 1989), with the inwardly rectifying K^+^ channel Kir6.2 (KCNJ11) and its accessory subunit SUR1 (ABCC8) playing a particularly central role (Gloyn et al., 2004; Hattersley and Ashcroft, 2005; Flanagan et al., 2009; Rubio-Cabezas et al., 2012). Together, Kir6.2 and SUR1 form in a 4:4 stoichiometry the K_ATP_ channel in pancreatic β-cells, which functions as a metabolic sensor (Ashcroft and Rorsman, 2013). At elevated blood glucose levels, the increasing glucose uptake by β-cells results in a high intracellular ATP:ADP ratio. This leads to an inhibition of the ATP-sensitive K_ATP_ channels, which in turn provokes a membrane depolarization and a subsequent opening of voltage-gated Ca^2+^ channels. The accompanying increase of intracellular Ca^2+^ levels then elicits increased insulin secretion (Figure 5).

Because of its prominent role in insulin secretion, the pancreatic β-cell K_ATP_ channel is a suitable target for modulating blood glucose regulation. Sulfonylureas such as glibenclamide and tolbutamide, for example, bind to SUR1 and inhibit the K_ATP_ channels, thereby increasing insulin secretion. Due to its opposite effect, the K_ATP_ channel activator diazoxide is used to counteract hypoglycemia. In general, sulfonylureas can be grouped into two generations of oral blood glucose-lowering medication. As the first-generation sulfonylureas are associated with a considerably higher risk of hypoglycemia and cardiovascular issues, these drugs have been replaced by improved (prolonged-release) second-generation sulfonylureas, which are associated with a lower risk of these adverse effects (Sola et al., 2015).

### 2.5 Glucose absorption by peripheral tissues

**FIGURE 6:**
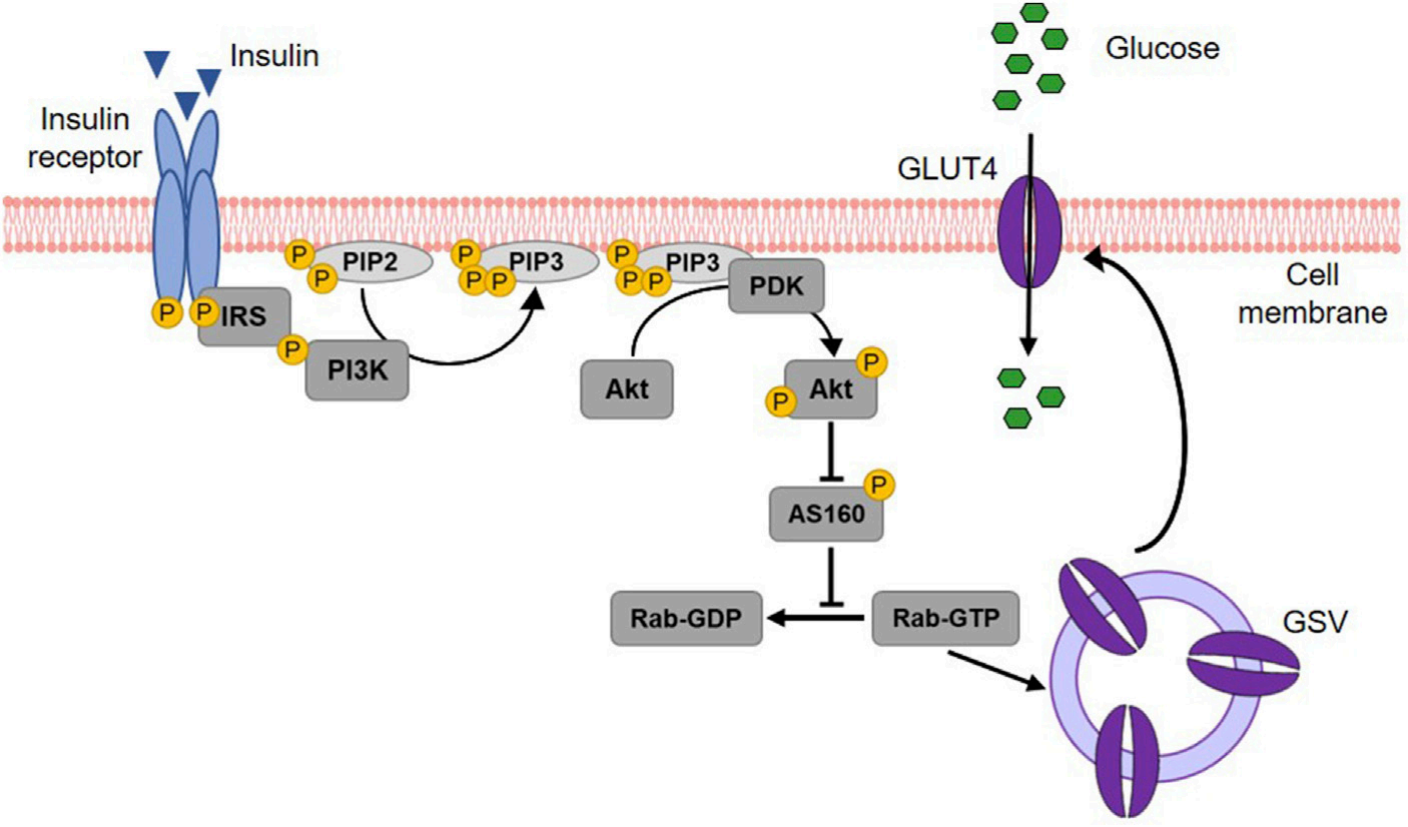
Insulin-stimulated GLUT4 translocation via the PI3K/Akt signalling cascade. Upon binding, insulin activates its receptor, which then phosphorylates and binds the insulin receptor substrate (IRS) protein. IRS binding recruits phosphoinositide 3-kinase (PI3K) to the cell membrane. PI3K phosphorylates membrane-bound phosphatidylinositol (4,5)-bisphosphate (PIP2) leading to the generation of phosphatidylinositol (3,4,5)-triphosphate (PIP3). This results in the recruitment of protein kinase B (Akt) to the membrane of the cell and the activation of phosphoinositide-dependent kinase (PDK) 1/2, which in turn phosphorylates Akt. Activated Akt subsequently phosphorylates the Akt substrate of 160 kDa (AS160), which thereby loses its GTPase activity. Consequently, Ras-associated binding (Rab) protein is activated as the hydrolysis of its bound GTP is prevented. Rab activation is responsible for the fusion of GLUT4 storage vesicles (GSV) with the cell membrane and thus for GLUT4 translocation. When integrated into the plasma membrane, GLUT4 finally enables the uptake of glucose into the cell (modified from (Thorn et al., 2013; Lankatillake et al., 2019; Heckmann et al., 2022). Used under CC BY 4.0: https://creativecommons.org/licenses/by/4.0/.

Insulin acts on three key target tissues to reduce blood glucose: liver, muscle and adipose tissue (Wilcox, 2005; Petersen and Shulman, 2018). While it suppresses endogenous glucose production and concurrently stimulates glycogen synthesis in the liver, insulin signaling promotes glucose uptake and metabolism in muscle and adipose tissue (Stadlbauer et al., 2020). Approximately 80% of postprandial rise in blood glucose levels is decreased by muscle tissue (Merz and Thurmond, 2020). The principal transporter responsible for glucose absorption by peripheral tissues is GLUT4, thus a key protein in whole-body glucose regulation (Huang and Czech, 2007). Since GLUT4 belongs to the facilitative glucose transporters, glucose is taken up into skeletal myocytes and adipocytes by passive diffusion following a concentration gradient (Satoh, 2014). The regulation of GLUT4-mediated glucose transport occurs primarily via its intracellular localization (Govers, 2014). In unstimulated (basal) cells, GLUT4 is retained in intracellular storage vesicles, while an increase of blood glucose level and subsequent insulin secretion triggers the translocation of GLUT4 to the plasma membrane (Govers, 2014; Lanzerstorfer et al., 2014). This process is mediated by the insulin signalling cascade (Kanai et al., 1993) illustrated in Figure 6. In brief, upon insulin binding, the tyrosine kinase activity of the insulin receptor phosphorylates the insulin receptor substrate protein (IRS), which in turn triggers the activation of phosphoinositide 3-kinase (PI3K) and the subsequent formation of phosphatidylinositol (3,4,5)-trisphosphate (PIP3) in the plasma membrane. PIP3 then recruits the protein kinase B (Akt) to the cell membrane, where Akt is phosphorylated and thereby activated by phosphoinositide-dependent kinase (PDK) 1/2 (Alessi et al., 1997; Stokoe et al., 1997; Dangelmaier et al., 2014). Active Akt suppresses the Akt substrate 160 (AS160) via phosphorylation, resulting in GTP loading of Ras-associated binding (Rab) proteins responsible for the initiation of GLUT4 translocation (Mîinea et al., 2005; Tan et al., 2012). Although PI3K/Akt is the generally recognized major signalling pathway leading to GLUT4 translocation, PI3K independent signals are also known to stimulate this trafficking event. In particular, the activation of the Rho family member GTPase TC10 via Cbl associated protein (CAP)/Cbl pathway, turned out to be a relevant factor for insulin-stimulated translocation of GLUT4 (Chiang et al., 2001; Watson et al., 2001; Chang et al., 2007).

## 3 Methodological toolbox to study glucose homeostasis modulating properties of plant extracts

### 3.1 *In-vitro* assays

#### 3.1.1 Enzyme assays for α-amylase, α-glucosidase and DPP4

**FIGURE 7:**
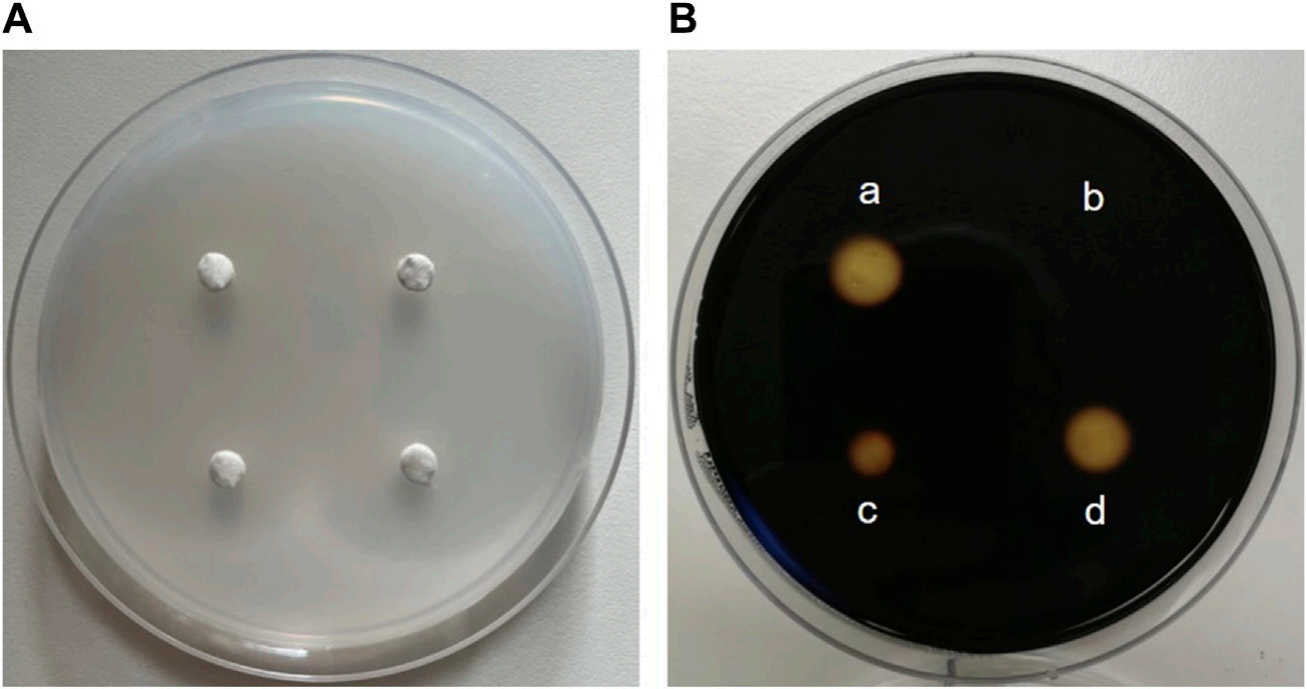
The disc assay for measuring inhibition of α-amylase activity. **(A)** Four “discs” made of filter paper with a diameter of 0.5 cm are placed on a Petri dish filled with medium (composed of 1% agar-agar and 1% starch). First, α-amylase (sourced from porcine pancreas) is added to solutions containing decreasing concentrations of an extract and pre-incubated, before the filter discs are soaked with the reaction mixture. The Petri plate is incubated at 37 °C for at least 8 h until the filter discs are removed. **(B)** Iodide-staining enables the calculation of the inhibition of enzyme activity. For this, the clear zone diameter of the control filter disc soaked with α-amylase alone **(a)** is compared with clear zone diameters of filter discs soaked with a solution of the extract and α-amylase (**b**, **c** and **d**). The stronger the inhibition of enzyme activity by the extract, the smaller the clear zone diameter.

The enzyme activity of α-amylase is mainly determined in two ways: with a disc diffusion method and spectrophotometrically. The disc assay is a method used for over 70 years to determine α-amylase activity (Goodman, 1950). It is based on α-amylase diffusing from a filter paper (disc) into a starch-containing agar gel, where it degrades the polysaccharide. Accordingly, a clear zone appears around the filter disc indicating the diffusion radius and the activity of the α-amylase when the undegraded starch in the plate is stained with iodine. The clear zone diameter can be determined, with the size of the diameter depending on the α-amylase concentration/activity (Briggs, 1962) (Figure 7). Thus, the disc assay is also applicable to study inhibition of the enzyme, e.g., by plant extracts. To this end, the α-amylase solution is mixed with the extract (preferably in various concentrations) before the filter discs are soaked (Correia et al., 2004). By comparing the diameter of the clear zone of a control sample (only enzyme solution) with those of the clear zone where the extract was added, the inhibition of α-amylase can be calculated. The assay is simple to perform and does not require expensive materials or equipment. In order to save material, downscaling of the protocol might be advantageous for screening approaches, thereby using 4 filter discs per agar-starch plate. A major advantage of this method is that the usually intense staining of plant extracts, which often interferes with the photometer-based assays discussed below, has no influence. However, due to its rather high inaccuracy, the disc assay is only suitable for general assessment of α-amylase inhibition by plant extracts.

A comprehensive protocol for spectrophotometrically determining α-amylase inhibition by plant extracts was developed by Apostolidis et al., 2006. After a porcine pancreatic α-amylase solution is pre-incubated with the plant extract, a solution of soluble starch is added as a substrate. The reaction incubated at 25 °C is stopped after 10 min by the addition of a colour reagent consisting of 3,5-dinitrosalicylic acid and sodium potassium tartrate and a 5 min incubation at 100 °C. The aromatic substance dinitrosalicylic acid reacts with the reducing sugars produced during starch degradation (Keharom et al., 2016). After cooling to room temperature, the absorbance of the reaction mixture, which reflects the α-amylase activity, can be measured at a wavelength of 540 nm (Apostolidis et al., 2006).

To determine the inhibition of α-glucosidase by plant extracts, a spectrophotometric assay is commonly used (Lankatillake et al., 2021). For this purpose, the enzyme is pre-incubated with the extract, followed by the addition of the chromogenic substrate p-nitrophenyl-α-D-glucopyranoside. The reaction incubated at 37 °C is terminated by the addition of a basic Na_2_CO_3_ stop solution, which also intensifies the yellow colour of the product p-nitrophenol. The α-glucosidase activity is proportional to the amount of p-nitrophenol released by the enzymatic reaction, the absorbance of which can be recorded spectrophotometrically at 405 nm (Lankatillake et al., 2021). For screening purposes, this assay can be easily performed in 96-well plate format, enabling a large number of samples to be run simultaneously.

Commercially available DPP4 inhibitor screening kits are suitable for high-throughput screening of putative DPP4 inhibitors. It can be performed in black 96-well microtiter plates according to the manufacturer’s instructions. The measurement is based on the cleavage of the substrate Gly-Pro-7-amido-4-methylcoumarin hydrobromide with yield of the fluorescence product 7-amido-4-methylcoumarin by active DPP4 enzyme (Kęska and Stadnik, 2022). The fluorescence signal (excitation/emission: 360/465 nm), which is proportional to the enzymatic activity present, can be measured over time.

For all assays, the percentage inhibition of the enzyme by an extract compared to a control can be determined. When performing the assay with suitable ascending extract concentrations, the half-maximal inhibitory concentration (IC_50_) (Aykul and Martinez-Hackert, 2016), can be calculated e.g., by using online IC_50_ calculator tools.

#### 3.1.2 Cell culture-based ussing chamber assay for sodium-dependent glucose transport

**FIGURE 8:**
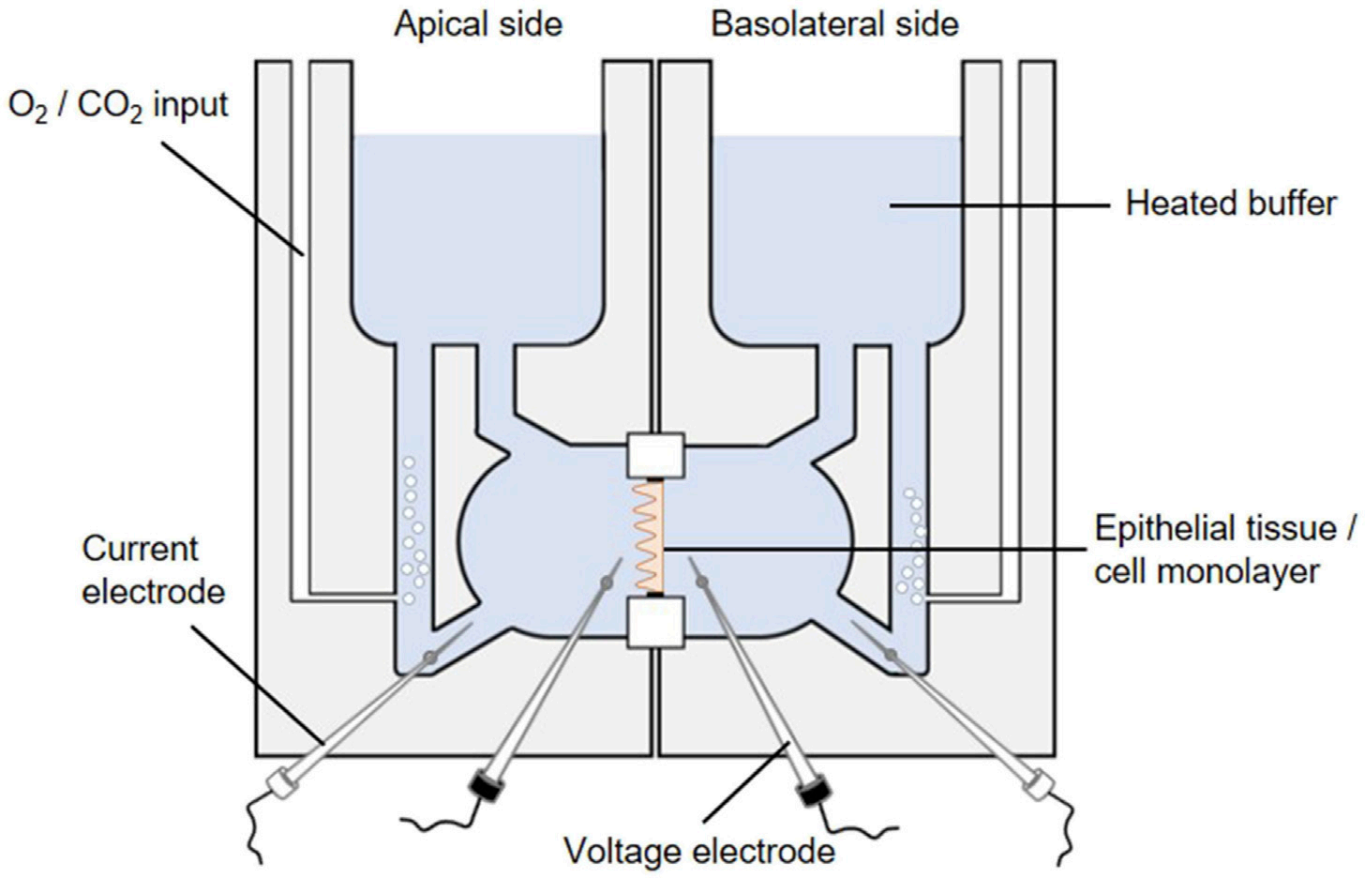
The Ussing chamber system. One Ussing chamber unit consists of two half-chambers, representing the apical and basolateral side, separated by a slider mounting an epithelial tissue or a cell monolayer. Both half-chambers are filled with equal volumes of the same 37 °C heated physiological salt solution. The solutions are oxygenated by carbogen gas while the temperature is maintained constant, so each side faces the same conditions. The transepithelial potential difference is continuously monitored through voltage electrodes (inner black electrodes). By using an automatic voltage clamp, the short-circuit current (I_SC_) is measured through current electrodes (outer white electrodes).

The activity of the SGLT1 transporter can be determined in cellular monolayers by employing the Ussing chamber. The Ussing chamber, developed by Hans Ussing in the 1950s (Larsen, 2002; Thomson et al., 2019), is a laboratory device that enables the measurement of substance fluxes such as ions, nutrients or drugs through epithelial tissues. The device (Figure 8) consists of two liquid-filled half-chambers separated by an epithelial tissue into an apical (mucosal) and basolateral (serosal) side, with substance flow between the two compartments via the epithelium. The liquid, a physiological buffer, is kept at a constant temperature of 37 °C and is continuously oxygenated with carbogen, a 95/5 (v/v) mixture of O_2_ and CO_2_. Moreover, by using identical electrolyte solutions of the same volume on both sides, there are no osmotic and electrochemical gradients either (Clarke, 2009). An inner pair of electrodes enables the measurement of the transepithelial potential difference between the apical and basolateral sides. Via an outer pair of electrodes, a short-circuit current (I_SC_) can be applied, which clamps this potential difference to 0 mV and reflects the actual active transepithelial ion transport (Clarke, 2009; He et al., 2013). This I_SC_ is recorded over time to monitor changes in transepithelial ion transport.

Different sliders available for an Ussing chamber system enables the variable use of epithelial tissues as well as Snapwell cell culture inserts comprising a permeable membrane supported by a detachable ring (Yeste et al., 2018). The intestine is one of the most intensively studied tissues in Ussing chambers in terms of gastrointestinal barrier function, permeability and nutrient absorption (He et al., 2013). Particularly, the active sodium and glucose co-transport via SGLT1 has mostly been studied by using animal tissue (Clarke, 2009; Schloesser et al., 2017; Klinger and Breves, 2018; Pallauf et al., 2019; Klinger, 2020). However, mounting Caco-2 (a human colorectal adenocarcinoma cell line) monolayers in Ussing chambers (Zheng et al., 2012; Steffansen et al., 2017) represent an intriguing alternative (Darling et al., 2020) to study plant extracts or compounds targeting the SGLT1-mediated glucose transport. Based on its properties, Caco-2 cells are a suitable *in-vitro* model for transport studies. Initiated by confluence, Caco-2 cells are starting to polarize and differentiate while developing an enterocyte-like phenotype, e.g., by forming a brush border and expressing specific brush border membrane enzymes (Hidalgo et al., 1989) and transporters for e.g., bile acids, amino acids and sugars including SGLT1 (van Breemen and Li, 2005). It should be pointed out that different Caco-2 clones exist distinguished by drastic differences in the expression of SGLT1. The highest SGLT1 expression is reported to be exhibited by the PD7 clone (Mahraoui et al., 1994).

For transport studies, Caco-2 cells are typically cultured for 21 days prior to use, as they have usually reached a fully differentiated state at this point (Darling et al., 2020). It has been proven effective to use the mucosal medium without serum supplementation after 7 days of cultivation, thereby simulating a more physiological condition and promoting the polarization process (Ferruzza et al., 2012). However, measurement of the transepithelial electrical resistance (TEER) as a marker of barrier tightness of a cellular monolayer is a suitable method to confirm the differentiation state of Caco-2 cells (Grasset et al., 1984).

Prior to Ussing chamber experiments the chambers are sealed with a blank slide and filled with the physiological buffer which is heated and oxygenated. Subsequently, adjustments are made to correct the offset potential between voltage-sensing electrodes and the fluid resistance. To start Ussing chamber experiments, the Snapwell inserts with the differentiated cell monolayer are transferred to Ussing chamber slides, before they are mounted into the prepared Ussing chamber. After refilling both half-chambers with the 37 °C physiological buffer, 10 mM glucose is added basolaterally as an energy substrate while 10 mM mannitol is added apically for osmotic reasons. The transepithelial potential difference is continuously recorded via Ag-AgCl electrodes and salt bridges (consisting of agar melted in the buffer used) (Clarke, 2009). Subsequently, the I_SC_ is measured via automatic voltage clamp (Clarke, 2009; Schloesser et al., 2017; Pallauf et al., 2019). By adding glucose to the mucosal side of the chamber (with simultaneous addition of mannitol to the serosal side) the sodium-coupled glucose transport is stimulated as indicated by elevated I_SC_ values. To determine SGLT1 inhibitory activity of plant extracts, they are added to both sides of the Ussing chamber and the reduction of the glucose-induced I_SC_ value is determined.

In general, it should be kept in mind that, although Ussing chamber studies provide an excellent opportunity to make accurate and rapid measurements of substance fluxes in a physiological context, the complexity of the physiological system is still not being mimicked to its full extent (He et al., 2013).

#### 3.1.3 Patch-clamp measurements for K_ATP_ channels

The potassium transport activity of the inwardly rectifying K^+^ channel Kir6.2, which is crucial for insulin secretion by pancreatic β-cells, can be determined by the patch-clamp technique. In the early 1950s, the concept of ion channels with conductances selective for different ions was proven by the fundamental work of Alan Hodgkin and Andrew Huxley (Hodgkin and Huxley, 1952b; 1952a). Thirty years later, the first genes coding for ion channels were identified and since then more than 400 ion channel genes have been cloned (Giraudat et al., 1982; Noda et al., 1982; Imbrici et al., 2016). In parallel, the patch-clamp technique has been developed (Hamill et al., 1981), which made it possible to examine the properties of the various ion channels after heterologous expression in a feasible expression system and to uncover their influence on cell physiology and thus also on physiology of the body.

**FIGURE 9:**
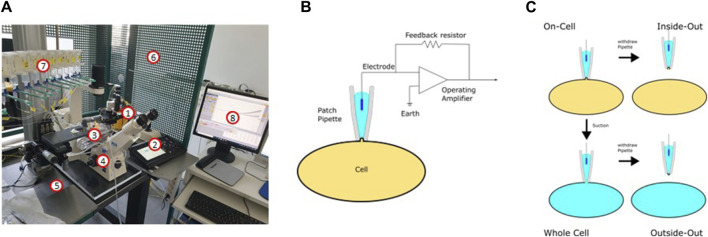
The patch-clamp technique. **(A)** Setup used for the patch-clamp measurements (1: headstage; 2: micromanipulator; 3: bath chamber; 4: microscope; 5: anti-vibration table; 6: Faraday cage; 7: application system; 8: software). **(B)** Electric circuit of the patch-clamp technique. **(C)** Different configurations of the patch-clamp technique.

Figure 9A depicts a typical set-up used for patch-clamp measurements. It is basically built around an inverse microscope placed on an anti-vibration table and surrounded by a Faraday cage. The heart of the setup is a preamplifier (probe or headstage) on which the patch pipette is mounted. The technique is based on the formation of a very tight contact of a glass pipette with the cell membrane (giga seal; Figure 9B). After the formation of the giga seal, the cell-attached configuration is achieved (Figure 9C), allowing the ion channels inside the patch to be measured without disturbing the intracellular milieu. Accordingly, the modulation of the channels by intracellular signal cascades can be evaluated. Starting from the cell-attached configuration, there are two possibilities to conduct measurements. One is to remove the membrane patch under the pipette by gentle suction obtaining the whole cell configuration. Here, all channels of the cytoplasma membrane contribute to the measurement, resulting in the largest currents being recorded. The cell is now filled with the defined pipette solution and the extracellular condition can be changed simply e.g., by adding drugs such as ion channel modulators and ligands of membrane receptors. The whole cell configuration is the standard configuration used when measuring the influence of modulators such as plant extracts on ion channel function. The second possibility is to withdraw the pipette from the cell-attached configuration. This leads to the inside-out configuration, where free access to the inside of the membrane is obtained. Consequently, the influence of pharmaka, changes in intracellular pH, fatty acids etc. can be measured in this configuration. From the whole cell configuration, you can also reach the outside-out configuration to investigate the channel without the intracellular milieu.

The patch-clamp technique can be used in two different modes: In the current clamp mode, the actual current seen by the cell can be either clamped to a certain value or no current at all is injected to the cell. In this mode, the potential between the intra- and extracellular milieu is measured. If the modulation of a channel leads to its opening or closing, this can be recorded as a change in the membrane potential or, in the case of excitable cells, as the generation of an action potential. In the current clamp mode, for example, the inhibition of inwardly rectifying K^+^ channels is noticeable as depolarization of the membrane, resembling the inhibition of the K_ATP_ channel in pancreatic β-cells after ATP elevation (Ashcroft and Rorsman, 2013). The second mode is the voltage-clamp mode. Here, the voltage is clamped to a certain value and the ionic current flowing through the membrane is measured as the counter current required to maintain the set voltage. Using the patch-clamp technique in combination with the preparation of slices of the organ of interest, every cell type in the body is available for electrophysiological measurements. Pharmacological investigations of ion channels are mainly done in the voltage clamp mode.

Most commonly used for the heterologous expression of ion channels are oocytes from *Xenopus laevis*, HEK293 cells, CHO cells or COS7 cells. These cell types have in common a relatively low background expression of endogenous ion channel genes. This allows the measurement of the respective channels without interfering endogenous currents. Following standard transfection protocols, plasmids for co-expression of Kir6.2 and SUR1 (e.g., available from Addgene; www.addgene.org/) are introduced into the cells and K^+^ channel function can be examined after 24 h already. The sulfonylurea drug glibenclamide is a suitable positive control having an IC_50_ value of about 1 µM (Houtman et al., 2021).

#### 3.1.4 Total internal reflection fluorescence microscopy for quantification of GLUT4 translocation

**FIGURE 10:**
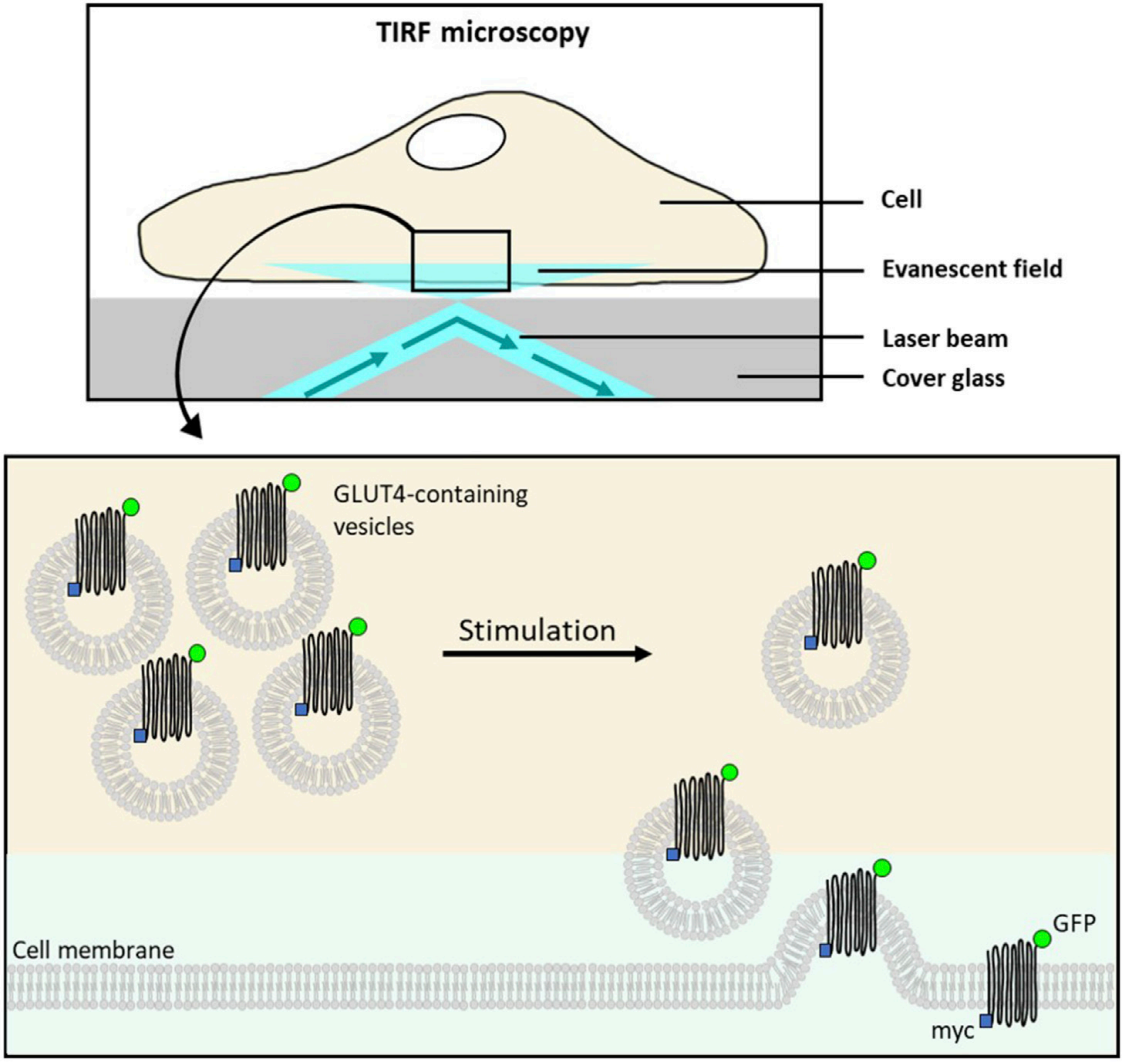
Schematic overview of TIRF microscopy for the quantification of GLUT4 translocation. The total internal reflection fluorescence (TIRF) microscopy is used to visualize fluorescent molecules located in an evanescent field very close to the interface between cover glass and water/specimen. This allows detection of membrane-associated proteins. The evanescent field is generated by a laser beam which is totally reflected at the interface. In unstimulated cells, stably expressing a glucose transporter 4 (GLUT4)-myc-green fluorescent fusion protein (GFP), GLUT4:GFP remains within the intracellular storage vesicles leading to the absence of fluorophores in the evanescent field. Insulin (or insulin activity mimicking plant extract/test compound) stimulation of cells results in the translocation and insertion of GLUT4-myc-GFP to the cell membrane. Consequently, the GFP signal intensity recoded by TIRF microscopy increases in the evanescent field (modified from (Lanzerstorfer et al., 2014; Stadlbauer et al., 2020)). Used under CC BY 4.0: https://creativecommons.org/licenses/by/4.0/.

The determination of GLUT4 translocation by total internal reflection fluorescence (TIRF) microscopy has been established as an excellent method for identifying drugs and plant extracts that can enhance GLUT4 translocation independently of insulin (Lanzerstorfer et al., 2014; Stadlbauer et al., 2016; Stadlbauer et al., 2020) (Figure 10). TIRF microscopy enables detection of fluorophores such as green fluorescent protein (GFP) located close to the interface between two media such as cover glass and sample and is characterized by extremely high sensitivity (Stadlbauer et al., 2020). Moreover, TIRF microscopy has been proven in several studies to be a suitable technique for screening approaches (Lanzerstorfer et al., 2014; Stadlbauer et al., 2016; Haselgrübler et al., 2018a; Stadlbauer et al., 2021). Nevertheless, it should be recognized that there are additional suitable methods for quantifying GLUT4 translocation, including microscopy-based approaches as well as biochemical and spectrometric approaches, as recently comprehensively reviewed (Heckmann et al., 2022).

To determine GLUT4 translocation modulating properties of substances or plant extracts, cell lines stably expressing a GLUT4-myc-GFP fusion protein are generated. Next to CHO-K1, HeLa turned out to be a suitable cell line, since no differentiation is required and the cells are characterized by good adhesion properties on various surfaces (Stadlbauer et al., 2020). In general, GLUT4-GFP expressing cells are grown in 96-well imaging plates, starved for at least 3 h and then treated with insulin (positive control), buffer (control) or test samples. Images can be taken at different time points before and after stimulation of the cells, using a microscope in TIRF configuration where the GFP fluorescence signal intensity correlates with GLUT4 accumulation at the cell membrane (Lanzerstorfer et al., 2014; Stadlbauer et al., 2016; Haselgrübler et al., 2018a; Stadlbauer et al., 2020; Stadlbauer et al., 2021). The analysis tool Spotty, which can be retrieved online (Research Group Bioinformatics Hagenberg, n. d.), provides e.g., an automated detection of fluorescent cells, the quantification of fluorescence signals or an automated calculation of signal changes (Lanzerstorfer et al., 2014), enabling increased throughput due to simplified data evaluation.

#### 3.1.5 Pitfalls and limitations of the *in-vitro* assays

Spectrophotometric and fluorometric assays are traditionally employed to quantify the inhibition potency of plant extracts against enzymes (Lankatillake et al., 2021). They are usually simple low cost assays suitable for high-throughput approaches. However, protocols often differ in some aspects e.g., enzyme concentration, incubation times or volumes used. Therefore, standardization of these assays should be attempted in order to improve comparability of results. To this end, the toolbox presented in this review might offer a valuable contribution. In general, limitations in terms of spectral interference from extract colour and turbidity should be noted. Therefore, blank-correction in spectrophotometric- and fluorometric-based enzyme assays is mandatory (Lankatillake et al., 2021). Considering that some plant extracts might exhibit autofluorescence, results should be interpreted critically with respect to possible false-positive hits (Stadlbauer et al., 2020). Moreover, the general use of enzymes of non-mammalian origin (Miller and Joubert, 2022) (e.g., α-glucosidase from *Saccharomyces cerevisiae* or porcine pancreatic α-amylase) and of non-natural substrates in enzyme screening assays may be additional pitfalls to be aware of.

Although the combination of Ussing chamber with Caco-2 cell culture has rarely been used to examine SGLT1-mediated glucose transport (Yin et al., 2014), recent publications show that this technique is excellent for studying the impact of extracts/substances on this target (Günther et al., 2021; Bauer et al., 2023; Lüersen et al., 2023). A particularly important parameter might be using the PD7 clone of the Caco-2 cell line (Mahraoui et al., 1994). However, it should be noted that the use of Caco-2 in monocultures has its limitations, including the lack of mucus, making co-cultures with mucin-secreting HT29 cells increasingly important (Béduneau et al., 2014). Another major disadvantage is that cytochrome P450 isozymes are poorly expressed in Caco-2 cells, resulting in a lack of metabolism of compounds during absorption process (van Breemen and Li, 2005). In this sense, it should be pointed out that *in-vitro* assays *per se* do not cover crucial aspects of pharmacology (Croston, 2017; Günther et al., 2021). Especially for target molecules located within the body such as Kir6.1/Sur2, Glut4 or DPP4, it should be borne in mind that high concentrations of plant extract-derived active ingredients may not be achieved *in-vivo*. On the other hand, the lack of biotransformation and the absence of a microbiota metabolism in *in-vitro* assays may prevent the formation of certain metabolites with putative bioactivity from plant extract ingredients.

The patch-clamp technique is ideally suited to characterize biophysical and pharmacological properties of certain ion channels after heterologous expression in the above-mentioned expression systems. If endogenous ion currents (for example, K_ATP_ channels in pancreatic β-cells) are to be measured, it is necessary to separate these currents from other endogenous ion currents. This can only be done successfully if specific inhibitors are available or if the biophysical properties can be clearly assigned to the channel under investigation. Furthermore, it is necessary to weigh up the patch-clamp-configuration used for the measurements: in cell-attached, inside-out and outside-out mode the currents are often very small and it must be ensured that the channels to be examined are in the patch; in whole-cell mode you have larger currents, but the intracellular solution is replaced by the pipette solution, so that intracellular signal molecules are washed out.

In general, when studying the bioactivity of plant extracts, one should be bear in mind that the solvent used for extraction can have a significant influence on the efficacy against individual targets (Jaradat et al., 2021), which is due to the fact that individual bioactive compounds dissolve differently depending on the solvent. However, even minor variations in the structure of putative bioactive compounds might significantly modify the effectiveness against a target. For example, studies postulate that structural characteristics such as the presence of hydroxyl groups in the B-ring and the C2-C3 double bond in the C-ring of flavonoids might be essential for α-glucosidase and DPP4 inhibition (Sarian et al., 2017; Li et al., 2018), while an additional Cl ion at 3-position of the C-ring might be particularly important for effective suppression of α-amylase (Proença et al., 2019). Moreover, Oranje et al. (2019) demonstrated that a substitution at the 9’ and 10’ C-atom of an angular pyranocoumarin backbone with an alkyl or alkene ester of 2–5 C-atoms was pivotal for SGLT1 inhibition. However, one should be aware that the effectiveness might not be necessarily due to a single compound and that synergistic modes of action can even be assumed as frequently discussed (Jaradat et al., 2024).

### 3.2 *In-vivo* assays

#### 3.2.1 Hen’s egg test

**FIGURE 11:**
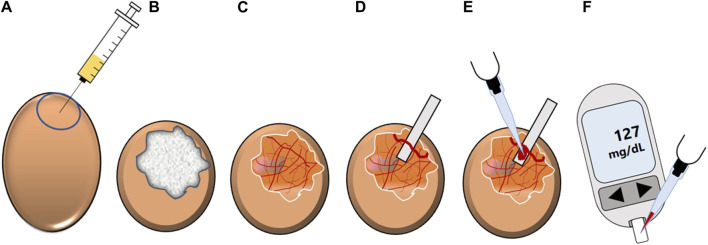
Schematic overview of the Gluc-HET experiment. **(A)** The location of the air bladder of a fertilized hen’s egg (11 days old) is marked and test substance-containing solution is applied into the air compartment. **(B)** The chorioallantoic membrane of the embryo is incubated with the solution. Afterwards, the eggshell is removed and the eggshell membrane is equilibrated. **(C)** After removal of the eggshell membrane, the chorioallantoic membrane is cut. **(D)** A main blood vessel is prepared on a pH-strip and **(E)** blood is collected from the vessel. **(F)** The blood glucose level is determined by using a blood glucose meter (modified from (Stadlbauer et al., 2021)). Used under CC BY 4.0: https://creativecommons.org/licenses/by/4.0/.

Blood glucose-reducing properties of synthetic compounds and plant extracts at an organismic level can be identified by a modified HET-CAM assay, the Gluc-HET test (Figure 11) (Haselgrübler et al., 2017; 2018b). The hen’s egg test (HET) is a well-established method for studying embryonic development, embryotoxicity, systemic toxicity, immunopathology and metabolic pathways (Luepke and Kemper, 1986). In general, the HET method offers advantages such as simple handling, acceptable costs and sufficient throughput rates (Haselgrübler et al., 2017; 2018b; Sandner et al., 2022, p. 20). An extended version of this test, the HET-chorioallantoic membrane (HET-CAM) test was developed as an alternative to the Draize rabbit eye test which is widely used in industry for evaluating membrane irritating properties of chemicals (Luepke and Kemper, 1986; Steiling et al., 1999; Haselgrübler et al., 2017). The CAM represents a highly vascularized extraembryonic membrane involved in gas exchange, acid-base homeostasis, calcium transport and reabsorption processes in the chicken embryo (Gabrielli and Accili, 2010). In brief, the HET-CAM assay is based on direct application of test chemicals onto the CAM of fertilized eggs and subsequent observation of adverse changes (Spielmann, 1995).

A similar strategy is applied in the Gluc-HET assay, where plant extracts or compounds diluted in buffer are injected into the air compartment of hen’s eggs and which are subsequently incubated for a defined time. This is followed by the preparation of the eggs for blood collection. To this end, the eggshell and, after an equilibration step, the eggshell membrane are removed, before the CAM can be uncovered to prepare a blood vessel on a pH-strip. Finally, blood collected from the vessel can be used to determine blood glucose levels (Haselgrübler et al., 2018b).

For this purpose, fertilized 10 or 11 day old eggs are used, with chicken embryos aged 11 days proving most suitable (Haselgrübler et al., 2017). This age provides several advantages. (i) Although not fully differentiated, the growth of the CAM during embryonic development is completed (Nowak-Sliwinska et al., 2014). (ii) Embryos at this age have high glucose levels (Haselgrübler et al., 2017). (iii) Insulin is produced by the embryos from day four or five, but the concentration of serum insulin is negligible until day 12, reducing the potential for interference between endogenously produced insulin and test substances. (iv) Insulin sensitivity is nevertheless already present at this stage (Yoshiyama et al., 2005; Haselgrübler et al., 2017). (v) The experiments are conducted at a stage in embryonic development when they are not considered as animal experiments (Haselgrübler et al., 2017; Sandner et al., 2022). These characteristics may make hen’s eggs a more attractive vertebrate model for determining the blood glucose-reducing properties of plant extracts than other vertebrate models such as the zebrafish, which is also frequently used as a model organism in the context of glucose homeostasis (Eames et al., 2010; Kakouri et al., 2020).

#### 3.2.2 *Drosophila melanogaster* models to evaluate the impact of plant extracts on glucose homeostasis and sugar-induced metabolic dysfunctions

The fruit fly *D. melanogaster* has proven to be a valuable model organism in nutrition research in recent decades (Staats et al., 2018; Lüersen et al., 2019; Eickelberg et al., 2022), especially due to the partly considerable similarity between human and *Drosophila* metabolism (e.g., approximately 65% of human disease-related genes having functional homologs in the fly) (Ugur et al., 2016). This also applies to the pathways of energy metabolism and glucose homeostasis in particular (Graham and Pick, 2017; Biglou et al., 2021; Chatterjee and Perrimon, 2021). Corresponding to the situation in humans, α-amylases and α-glucosidases are responsible for the intestinal degradation of poly- and disaccharides and GLUT transporters convey cellular glucose uptake. A sugar-responsive hormone neuropeptide F (NPF) acts similar to mammalian incretin-like hormones, and it is suggested that glucose is taken up via a sodium-dependent transporter (SLC5A5) in the midgut region (Li Y. et al., 2021). Moreover, *Drosophila* insulin-like peptides (Dilp), whose release from insulin producing cells (IPC) in response to rising glucose levels is mediated by a Kir6.1/SUR1-dependent mechanism similar to that in mammalian β-cells, counteract hyperglycaemia. Accordingly, the fruit fly has been found to be a suitable *in-vivo* model that can be employed not only to study and decipher molecular mechanisms of glucose homeostasis and related metabolic dysfunctions (Graham and Pick, 2017; Biglou et al., 2021; Chatterjee and Perrimon, 2021) but also to evaluate or verify modulating properties of plant extracts in this context (Bai et al., 2018; Günther et al., 2021; Nakitto et al., 2021; Ollinger et al., 2022; Bauer et al., 2023; Lüersen et al., 2023; Omoboyowa et al., 2023). This is underlined by the fact that established oral hypoglycemic agents such as acarbose (Günther et al., 2021), metformin (Abrat et al., 2018) and glibenclamide (Kréneisz et al., 2010) have also been found to be effective in *D. melanogaster*.

Being a genetic model in the first place, several *Drosophila* mutants and transgenes related to glucose homeostasis are available (Murillo-Maldonado and Riesgo-Escovar, 2017). Among these models, the *Ilp2* ^*1*^ *gd2HF* strain has been established expressing a double-tagged insulin-like peptide 2 (Ilp2HF), which allows quantification of circulating insulin in the fruit fly by an enzyme-linked immunosorbent assay (ELISA) (Park et al., 2014). Of the *Drosophila* Ilps, Ilp2 is essential for the maintenance of normoglycemia in flies (Grönke et al., 2010). The transgenetic flies were employed to monitor oral glucose stimulated insulin secretion in a fasting and re-feeding assay (Park et al., 2014). As recently demonstrated by Jans *et al.* (2024), the strain has also been proven to evaluate the impact of dietary supplements on levels of circulating postprandial Ilp2 in fruit flies. Further studies will reveal whether this transgene can also be used to screen plant extracts and bioactives with insulin secretion promoting properties.

**FIGURE 12:**
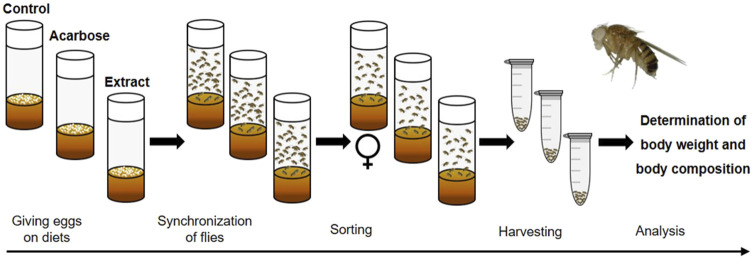
Schematic overview of the feeding experiment used in the *Drosophila melanogaster* model for dietary starch-induced obesity. Synchronized eggs are given in vials with a starch-based diet (control) or diets that were additionally supplemented with acarbose (positive control) or an extract (experimental diets). After eclosion, male and female flies are mated for 2 days. On day three, only mated females are transferred to fresh vials containing the respective diets. On day 10, the female flies are harvested. Subsequently, analyses are performed to determine the body weight and body composition of the flies (modified from (Eickelberg et al., 2022). Used under CC BY 4.0: https://creativecommons.org/licenses/by/4.0/.

Next to the genetic models, hyperglycemia and insulin resistance can be obtained through dietary approaches, feeding a high-sugar (HSD) or a high-fat diet (HFD) (Birse et al., 2010; Musselman et al., 2011; Morris et al., 2012). Similar to humans, this usually induces an obese phenotype associated with elevated triacylglyceride (TAG) levels. Notably, HSD feeding and a manifested diabetes-like status in the fruit fly have been shown to go along with long-term complications known from humans such as cardiopathy (Na et al., 2013) and nephropathy (dysfunction of the Malphigian tubules, the fruit fly kidney analog) (Na et al., 2015). Considering the conserved intestinal carbohydrate digestion enzymes, a dietary starch-induced obesity model was established to verify putative beneficial effects of plant extracts on glucose metabolism in a living organism (Figure 12). To this end, synchronized eggs are given onto a starch-based diet consisting of 10% soluble starch, 4% yeast and 1% agar according to Abrat et al. (2018). This diet is used either without supplement (control diet) or with acarbose given at 1.8 μg/mL (positive control) and plant extract (experimental diet), respectively. To avoid putative adverse developmental effects of the plant extracts, the treatment can be alternatively started in the adult stage on day 3 after eclosion. On day 10 after eclosion various readouts such as body weight and body composition can be determined (Eickelberg et al., 2022). These analyses are carried out on a sex-specific basis. For each group, the average body weight per fly is calculated by weighing 10–20 animals with a precision scale. For further analysis of TAG and protein content, a fly homogenate is made by grinding the flies in a buffer solution and subsequent centrifugation. Commercial kits are available for both parameters, which can be used according to the manufacturer’s instructions. If a reduction in the TAG level occurs in the treatment groups, this indicates that the glucose metabolism is targeted by the plant extract or compound.

Compared to rodents, this invertebrate model organism has numerous advantages. Due to an affordable maintenance, a high reproduction rate and a relatively short lifespan of *D. melanogaster*, high-throughput analyses are possible (Staats et al., 2018; Jans et al., 2021). Moreover, as the hen’s egg test, experiments can be carried out without serious ethical concerns and regulatory frameworks. Supplementation studies with *D. melanogaster* do not only cover most of the key features present in human carbohydrate metabolism but also many pharmacological aspects (e.g., bioavailability, biotransformation, role of gut microbiota, toxicity) (Günther et al., 2021; Lüersen et al., 2024). Despite this, physiological differences between the fruit fly and humans should be mentioned. In contrast to humans with one insulin hormone, the fruit fly possesses at least three Dilps involved in glucose homeostasis, which are expressed and secreted by neuroendocrine cells of the brain, as fruit flies lack a pancreas-like organ. Furthermore, trehalose rather than glucose is the predominant circulating sugar (Graham and Pick, 2017).

#### 3.2.3 Determination of glucose homeostasis modulating activity of plant extracts in rodents and humans

The evaluation of potential bioactive compounds or plant extracts in rodents and humans still represents the current gold standard, however obviously this is significantly more complex in terms of approval, time and ethical issues. There are numerous rodent models with impaired glycemic control, which are referred to as diabetes models. These are mainly mouse and rat models, which are thoroughly described in the literature (Chen and Wang, 2005, p. 200; Rees and Alcolado, 2005; Cefalu, 2006; Neubauer and Kulkarni, 2006; King, 2012; Kleinert et al., 2018; Kottaisamy et al., 2021), but none of these models reflect the pathological aspects of this disease entirely (Cefalu, 2006). In general, a phenotype with impaired glucose tolerance in rodents can be induced by chemical substances, such as alloxan (Dunn and McLetchie, 1943) and streptozotocin (Junod et al., 1969), or results from genetic alterations concerning, for example, leptin (*ob/ob* mice (Coleman, 1978)) or the leptin receptor (*db/db* mice (Coleman, 1978) and Zucker diabetic fatty rats (Peterson et al., 1990)). A high-fat diet represents an alternative to induce impaired glucose tolerance in these animals (Winzell and Ahrén, 2004; King, 2012; Calligaris et al., 2013).

Besides the determination of basal glucose and insulin levels, the glucose tolerance test, which monitors the change in blood glucose levels after glucose administration over time, is considered the standard method for assessing the impact of substances or plant extracts on glucose tolerance in rodents. The test can be varied considerably e.g., with respect to the applied glucose dosage, the route of its administration (oral, intraperitoneal injection or intravenous injection) or the expression of the results (absolute values vs. area under the curve) (Andrikopoulos et al., 2008; Bowe et al., 2014). However, the protocol as described here has proven useful in recent years to study the impact of dietary supplements in mice on glucose tolerance. After several weeks on an experimental diet (containing a supplement/plant extract), mice are fasted for 5–6 h prior to the glucose tolerance test. For this purpose, 2 g glucose/kg body weight is administered orally by gavage. Glucose levels are determined in blood taken from the tail tip by using a glucometer. Measurements are conducted prior to glucose administration and at several time points (ranging from 30 to 120 min) after administration (Schloesser et al., 2017; Fischer et al., 2020; Wüpper et al., 2020).

Similar to the glucose tolerance test, an insulin tolerance test, as indicator of insulin sensitivity, can be performed (Bowe et al., 2014). Moreover, a meal tolerance test is a suitable alternative in rodents (Manzano et al., 2016), which are thought to be more in a physiological context (Brodovicz et al., 2011). Despite the comparatively high effort (Muniyappa et al., 2008), some rodent studies using the glucose clamp technique to evaluate the antihyperglycemic activity of plant extracts can also be found (Manzano et al., 2016; Eddouks et al., 2017).

These methods are not only applied in animal models, but also find their implementation in human studies (Rijkelijkhuizen et al., 2009; Ajala et al., 2012; Besser et al., 2012; Lian et al., 2014; Vitetta et al., 2020). Clinical trials with plant extracts indicate that a variety of different parameters may reflect an antihyperglycemic activity in humans including HbA1c (glycated haemoglobin A1c) values, postprandial levels of glucose, insulin or C-peptide or the HOMA-IR (homeostasis model assessment of insulin resistance) index (Ahmad R. et al., 2021; Lim et al., 2021). Since its development in 1979 (DeFronzo et al., 1979), the glucose clamp technique has emerged as the most appropriate method to evaluate potential antidiabetic agents in humans (Tam et al., 2012; Pelegrin et al., 2019). Depending on the specific issue, either the hyperglycemic clamp technique, as an indicator of islet cell functionality, or the euglycemic insulin clamp technique, as an indicator of insulin sensitivity, can be employed. Both methods rely on the use of variable glucose infusion rates administered to probands to maintain (clamp) blood glucose at a constant level following an initial glucose bolus (hyperglycemic clamp) or a continuous insulin infusion (euglycemic insulin clamp) (DeFronzo et al., 1979).

## 4 Plant root extracts with putative impact on key regulatory elements of glucose homeostasis

Based on their traditional use against various metabolic diseases such as diabetes, the *in-vivo* blood glucose-lowering potential of medicinal plants and plant extracts is widely reported for rodent models or humans (Bailey and Day, 1989; Eddouks et al., 2017). As discussed above, the use of root extracts, which have received little attention so far, is of particular interest. In this context, Ardalani et al. (2021) recently summarized hyperglycemia-reducing properties of root and rhizome extracts derived from 104 plant species in rodent models, but the mechanism of action commonly remains unclear. However, although mechanistic investigations concerning the impact of root and rhizome extracts on key regulatory elements of glucose homeostasis are rare, some studies address this particular issue. They are summarized in Table 1. For this purpose, studies which are published after 2002, were searched via scientific databases using the targets of glucose homeostasis as search terms in combination with the keyword “root extract(s).” Studies were excluded when extracts without a defined solvent were used.

**TABLE 1:** Overview of root and rhizome extracts evaluated for their impact on key regulatory elements of glucose homeostasis.

Plant species	Traditional use	Solvent	Molecular target		Main compounds putatively responsible for bioactivity	References
			*In-vitro*	*In-vivo*		
*Allium hookeri* Thwaites	For foods and medicine in Asia	Water	Increased GLUT4 expression and glucose uptake in 3T3-L1 adipocytes	-	-	Yang et al. (2016)
*Ampelopsis japonica* (Thunb.) Makino	Remedy for fever, pain, and wound healing	Hexane; Dichloromethane; chloroform; acetone; Ethyl acetate; ethanol; Methanol; Water	Inhibition of α-glucosidase		Catechin, kaempferol, quercetin, euscaphic acid, resveratrol, epicatechin	Liang et al. (2022)
*Annona stenophylla* Engl. & Diels	Treatment of various aliments in Zimbabwe	Water	Increased GLUT4 expression, GLUT4 translocation and glucose uptake in C2C12 muscle cells	-	-	Taderera et al. (2019)
*Aristolochia ringens* Vahl	Management of DM in Nigeria by Traditional Medicine Practitioners	Ethanol (70%)		Reduction of α-amylase activity in the liver of diabetic rats	Aristolone	Sulyman et al. (2016)
*Aristolochia ringens* Vahl	Various medicinal applications in West African countries	Ethanol (70%)	Inhibition of α-amylase and α-glucosidase		Dianoside G, trilobine, asiatic acid, magnoflorine, quercetin 3-O-glucuronide, strictosidine	Ahmad et al. (2021a)
*Atractylodes japonica* Koidz. Ex Kitam.	Treatment of obesity and related complications in East Asia	Water	Promoted glucose transport by increasing GLUT4 expression, PI3K and IRS-1 levels	-	-	Han et al. (2012)
*Bistorta officinalis* Delarbre	Medicine against various medical conditions (e.g., gastrointestinal disorder, haemorrhoids, snakebites)	Water	Inhibition of α-amylase, α-glucosidase, SGLT1 and DDP4	-	Gallotannins, catechins, chlorogenic acid, galloyl slidroside	Bauer et al. (2023)
*Canna indica* L.	Application in Thai folkloric medicine to treat DM	Water	Increased GLUT4 translocation and glucose uptake in L8 muscle cells	-	Flavonoids, catechol	Purintrapiban et al. (2006)
*Capparis spinosa* L.	Treatment of different health problems like hyperglycemia in Central Asia; Control of DM in Marocco	Methanol; Dichloromethane	Inhibition of α-amylase and α-glucosidase	-	Glucosinolates, alkaloids, flavonoids, phenols, triterpenes, alkaloid derivatives	Saleem et al. (2021)
*Cajanus cajan* (L.) Huth	Use as anthelminthic, alexeritics and expectorant by Taiwanese aborigines	Ethanol (95%)	Inhibition of α-amylase and α-glucosidase	-	-	Yang et al. (2022)
*Cissus cornifolia* (Baker) Planch.	Treatment of DM in Zimbabwe	Ethanol; Water	Inhibition of α-amylase and α-glucosidase	-	-	Chipiti et al. (2017)
*Curculigo latifolia* Dryand. ex W.T.Aiton	-	Water	Inhibition of α-glucosidase and DPP4; increased glucose uptake and insulin secretion	-	Phlorizin	Zabidi et al. (2021)
*Curcuma longa* L.	Household remedy used for multiple disorders	Ethyl acetate	Inhibition of α-glucosidase and DPP4	-	-	Inbaraj and Muniappan (2020)
*Curcuma longa* L.	Used as food color and preservative in Asian Countries	Ethanol	Inhibition of α-glucosidase		Demethoxycurcumin, isorhamnetin, valoneic acid bilactone, curcumin, curcumin-O-glucuronide	Sabir et al. (2021)
*Curcuma manga* Valeton & Zijp	Native Indonesian medicinal plant associated with many beneficial effects	Ethanol (70%)	Increased GLUT4 expression and glucose uptake in 3T3-L1 adipocytes	-	-	Pujimulyani et al. (2020)
*Dioscorea batatas* Decne.	Treatment of various disorders such as DM in Asian traditional medicine	Water		Induction of GLP-1 in streptozotocin-induced diabetic rats	Allantoin	Go et al. (2015)
*Gentiana scabra* Bunge	Remedy for DM in traditional Korean medicine	Water	Induction of GLP-1 secretion in NCI-H716 cells	Increased plasma GLP-1 level after glucose load in *db/db* mice	Loganic acid	Suh et al. (2015)
*Geum urbanum* L.	-	Water	Inhibition of α-glucosidase but not α-amylase; Inhibition of SGLT1 and DPP4; Induction of GLUT4 translocation	-	Ellagic acid (derivatives), ellagitannins	Günther et al. (2021)
*Hedychium coronarium* J. Koenig	Treatment of various disorders in Vietnam and traditional Indian medicine	Ethyl acetate	Inhibition of α-amylase and α-glucosidase		Suberic acid, triparanol, ginkgolide C, swietenine	Panigrahy et al. (2020)
*Limonium axillare* (Forssk.) Kuntze	Remedy for DM in Eastern Egypt	Ethanol (70%)	Inhibition of α-amylase and α-glucosidase	Reestablished GLUT2 and GLUT4 expression, amelioration of pancreatic tissue damage in streptozotocin-induced diabetic rats	Umbelliferone, tetrahydrofuran monoterpenes (2-isopropyl- 3,4,4, trimethyl-tetrahydrofuran and 2-isopropyl-4-methyl-tetrahydrofuran-3,4dicarboxylic acid)	Abdel-Sattar et al. (2021)
*Limonium bellidifolium* (Gouan) Dumort	Treatment of various disorders (e.g., fever, diarrhea) in Mediterranean region	Methanol	Inhibition of α-amylase but not α-glucosidase	-	-	Senizza et al. (2021)
*Limonium globuliferum* (Boiss. & Heldr.) Kuntze			Inhibition of α-amylase but not α-glucosidase	-	-	
*Limonium gmelinii* (Willd.) Kuntze			Inhibition of α-amylase, weak inhibition of α-glucosidase	-	-	
*Limonium iconicum* (Boiss. & Heldr.) Kuntze			Inhibition of α-amylase but not α-glucosidase	-	-	
*Limonium lilacinum* (Boiss. &balansa) Wagenitz			Inhibition of α-amylase, weak inhibition of α-glucosidase	-	-	
*Limonium sinuatum* (L.) Mill.			Inhibition of α-amylase but not α-glucosidase	-	-	
*Molineria latifolia* (Dryand. Ex W.T.Aiton) Herb. Ex Kurz	-	Methanol (80%)	Increased GLUT4 translocation and glucose uptake	-	Curculigoside	Ooi et al. (2018)
*Moltkia aurea* Boiss.	Treatment of various health problems (e.g., diarrhea, abdominal pain) in eastern part of Mediterranean region	Water; Methanol; Ethyl acetate	Inhibition of α-glucosidase but not α-amylase	-	Rosmarinic acid	Orhan et al. (2021)
*Moltkia coerulea* (Willd.) Lehm	Treatment of various health problems (e.g., diarrhea, abdominal pain) in eastern part of Mediterranean region	Water; Methanol; Ethyl acetate	Inhibition of α-glucosidase but not α-amylase	-	Rosmarinic acid	Orhan et al. (2021)
*Morus alba* L.	Treatment of various disorders such as DM in the Chinese Pharmacopoeia, Korea and Japan	Ethyl acetate	Inhibition of α-amylase and α-glucosidase	-	Diels-Alder adducts, isoprenylated flavonoids	Zhao et al. (2018)
*Morus alba* L.	Treatment of chronic disorders, especially by the Chinese Pharmacopoeia	Ethanol	Inhibition of α-glucosidase	-	Stereoisomers of dicyclokuwanon EA	Tian et al. (2024)
*Morus mesozygia* Stapf.	Treatment of inflammatory conditions in West Africa	Methanol (80%)	Inhibition of α-glucosidase and DPP4 but not α-amylase	-	7-(3-hydroxy-3-methylbutyl)moracin M, moracin P and moracin M	Olufolabo et al. (2024)
*Myrica salicifolia* Hochst. Ex A.Rich.	Treatment of DM related aliments in Ethiopia and Tanzania	Methanol (80%)	Inhibition of α-amylase	-	-	Emiru et al. (2020)
*Paeonia anomala* L.	-	Methanol	-	Inhibition of α-amylase in isolated plasma of mice	-	Kobayashi et al. (2003)
*Peucedanum praeruptorum* var. grande K.T.Fu	Traditionally been indicated for treatment of obesity and hyperglycemia	Methylene chloride	Inhibition of uptake and transport of 14C-α-methylglucose by Caco-2 cells expressing endogenous SGLT1	-	(+)-Pteryxin	Oranje et al. (2019)
*Picrorhiza kurroa* Royle ex Benth.	Remedy for DM in traditional Indian medicine	Water	-	Increased GLUT4 translocation and glucose uptake in streptozotocin-induced diabetic rats	Kutkin	Husain et al. (2014)
*Polygonum cuspidatum* Siebold & Zucc.	Traditional antidiabetic herb used in China; Functional food in Japan and South Korea	Ethyl acetate	Inhibition of α-amylase and α-glucosidase	-	Procyanidin B2 3,3″-O-digallate, (−)-epicatechin gallate, stilbene analogues	Zhao et al. (2017b)
*Polygonum viviparum* L.	Treatment of diarrhea and blood stasis in China	Methanol (60%)	Inhibition of α-glucosidase	-	Quercetin−3−O−vicianoside, quercetin 3−O−neohesperidoside, rutin, hyperoside, quercetin 3−O−glucuronide, luteolin−7−O−neohesperidoside, and kaempferol 3−glucuronide	Li et al. (2021a)
*Pueraria tuberosa* (Roxb. Ex Willd.) DC.	Used as hypoglycemic drug in traditional Indian medicine	Water	Inhibition of DPP4	Inhibition of DPP4 and increased GLP-1 concentration in plasma of rats	-	Srivastava et al. (2015)
*Pueraria tuberosa (Roxb. Ex Willd.)* DC.	Used as hypoglycemic drug in traditional Indian medicine	Water	-	Induction of GLP-1 and GLP-1R expression in rats	Tuberostan and puererone	Srivastava et al. (2018)
*Rehmannia glutinosa* Libosch	Treatment of various disorders (e.g., DM, haematological conditions, insomnia) in traditional Chinese medicine	Methanol (100%, 80%, 60%); ethanol (100%, 80%, 60%); Ethyl acetate; acetone; Water	Inhibition of α-glucosidase	-	-	Jeong et al. (2013)
*Rhazya stricta* Decne.	Treatment of DM in traditional medicine systems	Ammonical-chloroform:methanol (1:1)	Inhibition of α-glucosidase and DPP4; induction of GLP-1 secretion in cultured cells	-	-	Mahmood et al. (2020)
*Rhodiola crenulata* (Hook.f. & Thomson) H. Ohba	Treatment of various disorders (e.g., fatigue, asthma) in Tibet and China	Methanol	Inhibition of α-amylase and α-glucosidase	Reduction of postprandial glucose levels of mice in a starch tolerance test	Hydroxybenzoic acids, hydroxycinnamic acids, alcohol glycosides, flavonols and their derivatives	Yue et al. (2022)
*Rhodiola rosea* L.	-	Methanol	-	Inhibition of α-amylase in isolated plasma of mice	-	Kobayashi et al. (2003)
*Rhodiola rosea* L.	Treatment of fatigue and depression and improvement of physical activity in northern latitudes	Water		Increased GLUT4 expression in skeletal muscle in streptozotocin-induced diabetic rats	Salidroside, p-tyrosol	Niu et al. (2014)
*Rhodiola rosea* L.	-	Water	Inhibition of α-amylase, α-glucosidase, SGLT1and DPP4	-	Rosarin, gallic acid	Günther et al. (2021)
*Salacia oblonga* Wall. ex Wight & Arn.	Treatment of DM in traditional Indian medicine	Methanol (80%)	Inhibition of α-amylase and α-glucosidase	-	-	Deepak et al. (2020)
*Salvia ceratophylla* L.	-	Hexane; Dichloromethane; Methanol; Methanol (80%); Water	Inhibition of α-amylase and α-glucosidase	-	-	Uysal et al. (2021)
*Securidaca longepedunculata* Fresen	-	Assay buffer	Inhibition of α-amylase	-	-	Funke and Melzig (2006)
*Smilax aristolochiifolia* Mill.	Traditionally used as hypoglycemic and for weight loss in Mexico	Ethanol (50%)	Inhibition of α-amylase	-	Chlorogenic acid, astilbin	Pérez-Nájera et al. (2018)
*Solanum incanum* L.	Treatment of DM in Ethiopian folkloric medicine	Methanol (80%)	Inhibition of α-amylase	-	-	Andargie et al. (2022)
*Stereospermum tetragonum* DC.	-	Water	Increased GLUT4 expression in A431 cells		1,4a,5,7a-Tetrahydro-5hydroxy-7-(hydroxymethyl)-1-(tetrahydro-6-(hydroxymethyl)-3,4,5-trimethoxy-2H-pyran-2-yloxy)cyclopenta [c]pyran-4-carboxylic acid and 5,8-dihydro-7isopentyl-2,3,5,8-tetramethoxynaphthalene-1,4,6-triol	Kingsley et al. (2014)
*Strophanthus hispidus* DC.	Treatment of various disorders (e.g., inflammatory conditions, DM) in Africa	Water	Inhibition of α-amylase and α-glucosidase	-	-	Fageyinbo et al. (2019)
*Synclisia scabrida* Miers	Remedy for various disorders (e.g., abdominal pains, mental strain, DM) in several African regions	Methanol	Inhibition of α-amylase and α-glucosidase	-	-	Orumwense et al. (2022)
*Taraxacum officinale* F.H.Wigg.	Traditional medicine and food used worldwide	Water	Inhibition of α-amylase and α-glucosidase	-	-	Li et al. (2021b)
*Uvaria chamae* P.Beauv.	Treatment of DM in West Africa	Ethanol (93.3%)	Inhibition of α-amylase and α-glucosidase	-	-	Emordi et al. (2018)
*Vernonia amygdalina* Delile	Treatment of hyperglycemia in Africa	Methanol	Inhibition of α-amylase and α-glucosidase	-	-	Igbinidu and Nimenibo Uadia (2019)
*Vernonia amygdalina* Delile	Treatment of various disorders (e.g., hypertension, malaria, DM) in African medicine	Water	Inhibition of α-glucosidase	-	-	Medjiofack Djeujo et al. (2021)
*Vitex agnus*-*castus* L.	Treatment of various disorders (e.g., premenstrual syndrome, stomach ache, DM)	Methanol	Inhibition of α-amylase and α-glucosidase	-	-	Berrani et al. (2021)
*Withania somnifera* (L.) Dunal	Versatile use in traditional Indian medicine	Methanol	Inhibition of DPP4	-	Catechin	Kempegowda et al. (2018)
*Zingiber officinale* Roscoe	Widely used in traditional medicines worldwide	Ethyl acetate	Increased GLUT4 expression and glucose uptake in L6 cells	-	Gingerol and shogaol	Rani et al. (2012)

Only studies are listed in which an extract with defined solvent was tested for one or more of the targets α-amylase, α-glucosidase, sodium-dependent glucose transporter (SGLT1), dipeptidyl peptidase 4 (DPP4), glucagon-like peptide-1 (GLP-1) or glucose transporter 4 GLUT4). Diabetes mellitus (DM).

Almost all of the plant species listed in Table 1 share a significant ethnopharmacological background, which is also referred to in the table. For example, with plant species such as *Hedychium coronarium* J. Koenig (Panigrahy et al., 2020), *Pueraria tuberosa* (Roxb. Ex Willd.) DC. (Srivastava et al., 2015), *Salacia oblonga* Wall. ex Wight & Arn (Deepak et al., 2020). and *Withania somnifera* (L.) Duna (Kempegowda et al., 2018), reference is made to their intensive use in traditional Indian medicine/Ayurveda. In African folk medicine *Annona stenophylla* Engl. & Diels (Taderera et al., 2019), *Aristolochia ringens* Vahl (Sulymean et al., 2016), *Cissus cornifolia* (Baker) Planch. (Chipiti et al., 2017), *Myrica salicifolia* Hochst. Ex A. Rich. (Emiru et al., 2020), *Solanum incanum* L. (Andargie et al., 2022) or *Vernonia amygdalina* Delile (Medjiofack Djeujo et al., 2021) are employed for the treatment of various health problems while in the Chinese Pharmacopoeia the use of plant species such as *Dioscorea batatas* Decne. (Go et al., 2015), *M. alba* L. (Zhao et al., 2018), *Polygonum viviparum* L. (Li H. et al., 2021) or *Rehmannia glutinosa* Libosch (Jeong et al., 2013) is prominent. Basically, the studies with the plant root extracts listed were set up for various reasons. (i) The studies attempt to find a scientific rationale for the traditional use of the plant species studied in the treatment of diabetes. (ii) The studies are intended to elucidate the underlying molecular mechanisms responsible for the demonstration of antihyperglycemic effects of the plant roots in an *in-vivo* rodent model. (iii) A screening approach, for example, using a selection of Mongolian plants (Kobayashi et al., 2003) or using a plant extract library (Günther et al., 2021), was carried out with respect to one of the targets of glucose homeostasis to identify new bioactive plant material.

Approximately 50 plant root extracts have been described with respect to their activity toward α-amylase, α-glucosidase, SGLT1, GLP-1, DPP4 and GLUT4. The most abundant targets evaluated in these studies are the carbohydrate-hydrolysing enzymes α-amylase and α-glucosidase followed by GLUT4. Interestingly, a comparison of IC_50_ values indicated that an ethyl acetate extract of *M. alba* L. was the most potent inhibitor of both α-amylase (IC_50_ value of 5.16 μg/mL) and α-glucosidase (IC_50_ value of 1.70 μg/mL), while, for example, root extracts from various *Limonium* species were found to specifically inhibit the α-amylase (Senizza et al., 2021) and root extracts from different *Moltkia* species or from *Geum urbanum* L. were found to specifically inhibit the α-glucosidase (Günther et al., 2021; Orhan et al., 2021). There are only few studies investigating the modulating activity of plant root derived extracts on the incretin system (DPP4, GLP-1) and on SGLT1. However, concerning the incretin system, the aqueous extract of roots from *P. tuberosa* (Roxb. Ex Willd.) DC. might be of interest as a modulation of DPP4 and GLP-1 was demonstrated both *in-vitro* and *in-vivo* (Srivastava et al., 2015).

Besides conventional targets (α-amylase, α-glucosidase, SGLT1, GLP-1, DPP4 and GLUT4), whose modulation by root extracts has been investigated so far, inhibition of KATP channels could be an emerging focus. Modulation of pancreatic insulin secretion by plant extracts and their phytochemicals has already been demonstrated and might be relevant for future (Govindappa, 2015; Hager et al., 2021). For example, a high-content screen of aqueous plant extracts led to the identification of several potential modulators of glucose-stimulated insulin secretion in living MIN6 β-cells by using an insulin-Gaussia luciferase biosensor (Hager et al., 2021). In this context, it could be particularly interesting to examine the influence of root extracts on pancreatic β-cell KATP channels via patch-clamp technique.

Recent results of studies that have systematically investigated several of the above-mentioned targets indicate that plant (root) extracts indeed have the potential to influence more than one of the key regulatory elements of glucose homeostasis simultaneously, as demonstrated by the example of a *G. urbanum* L. root extract (Günther et al., 2021), a *Bistorta officinalis* Delarbre root extract (Bauer et al., 2023) and a *Morus mesozygia* Stapf. root bark extract (Olufolabo et al., 2024). In this context, we are convinced that the systematic investigation of the key elements of glucose homeostasis could also be of further interest for the other promising root extracts listed in Table 1 and that the toolbox presented here could considerably facilitates this approach. Moreover, it should be noted that it might be highly beneficial to use a combination of two or more plant roots (extracts), as this combination could be significantly more effective by modulating different mechanisms of glucose homeostasis simultaneously. Fundamental scientific findings in this context could also be beneficial to traditional medicines. Modern biomedical science continues to explore the ethnobotanical value of roots to validate traditional knowledge and discover novel applications. Thus, integrating traditional knowledge with modern scientific research technologies holds great promise for discovering new therapeutic agents and improving public health, as exemplified by metformin, which originated from the traditional used *Galega officinalis* L. (Bailey, 2017). Accordingly, ethnobotanical research often focuses on isolating active compounds, understanding their underlying cellular and molecular mechanisms of action, and conducting clinical trials to confirm their efficacy and safety in humans.

## 5 Summary and perspectives

**FIGURE 13:**
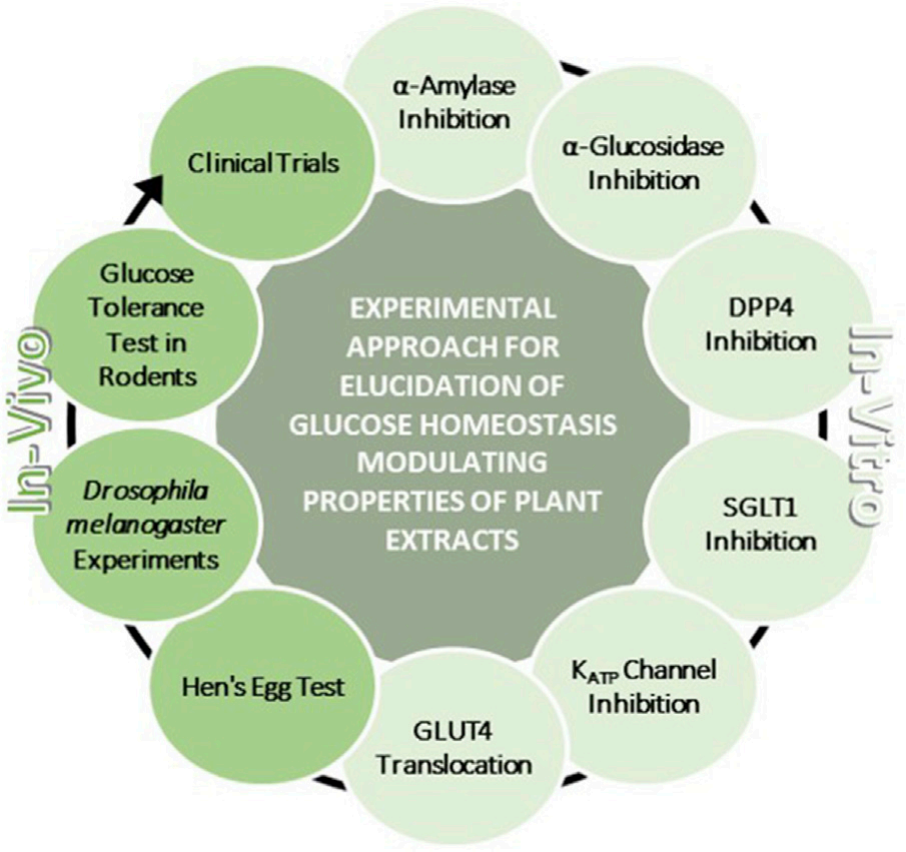
Schematic overview of a methodological approach to systematically study glucose homeostasis modulating properties of plant extracts. This approach comprises *in-vitro* studies in terms of α-amylase inhibition, α-glucosidase inhibition, dipeptidyl-peptidase 4 (DPP4) inhibition, sodium-dependent glucose transporter 1 (SGLT1) inhibition, K_ATP_ channel inhibition and glucose transporter 4 translocation. Also included are *in-vivo* studies in model organisms, namely, chicken embryos, *Drosophila melanogaster* and rodents, and also in humans.

Natural substances, and plant extracts in particular, already play an important role in the control of glucose homeostasis. It is therefore promising to expand our portfolio of natural substances in this field and to find even more effective drugs with fewer side effects. This also applies in particular to compounds from plant roots, which have been little investigated to date. The growing importance of medicinal plant extracts as a source of blood glucose-regulating medications increases the demand for a systematic experimental approach to screen larger amount of plant extracts, such as those generated e.g., when employing extract libraries and sub-libraries, against multiple targets of glucose homeostasis and to evaluate promising extracts in appropriate *in-vivo* models and humans. The corresponding workflow proposed in this review (Figure 13) is intended to represent such a roadmap starting with enzyme inhibition assays suitable for (high-throughput) screening-approaches, through *in-vivo* models suitable for intermediate screenings such as the hen’s egg assay or the *D. melanogaster* models, up to complex experiments concerning the blood glucose-lowering potential of plant extracts in rodents and humans.

From the previous studies on plant (root derived) extracts with blood glucose-lowering activity, two principal weaknesses can often be deduced. First, based on their traditional use, the blood glucose-lowering potential of medicinal plants and plant extracts is directly demonstrated *in-vivo* in diabetic rodents or humans, but the mechanism of action usually remains unclear. Second, when plant extracts were tested in *in-vitro* assays, only selected targets of glucose homeostasis are commonly studied, rather than systematically examining a broader range of key regulatory elements of glucose homeostasis. With respect to plant extracts, this is particularly important, as a pleiotropic effect can certainly be assumed. Thus, the application of the presented toolbox allows to cover many important steps of glucose homeostasis in a rational way and may complement promising study results available so far on root extracts as listed in Table 1. Our own studies in recent years, in which the majority of the assays included in this toolbox were systematically applied, revealed that plant extracts have the potential to influence several key elements of glucose homeostasis simultaneously and demonstrated the relevance of combining *in-vitro* assays and (relatively simple) *in-vivo* models when evaluating the putative bioactivity of plant extracts in this context (Günther et al., 2021; Bauer et al., 2023). Subsequent steps could focus on counter-current fractionation and bioassay-guided separation to identify the bioactive compounds.

However, further expansion of the toolbox may be worthwhile, for example, the investigation of the impact of plant extracts on incretin hormones such as GLP-1, by determining GLP-1 secretion in cultured enteroendocrine GLUTag cells (Esatbeyoglu et al., 2016), thereby complementing DPP4 data. Moreover, GLUT2 represents a promising target, since it was shown that GLUT2 is translocated to the apical membrane of enterocytes under high-glucose concentrations (Zheng et al., 2012; Müller et al., 2018). Likewise, inhibition of the enzyme protein tyrosine phosphatase 1B (PTP1B) by plant extracts is getting increasingly more attention (Zhao Y. et al., 2017; 2018; Sun et al., 2019). As a negative regulator of insulin receptor signaling, PTP1B inhibition may lead to improved insulin sensitivity and/or increased glucose uptake (Elchebly et al., 1999; Klaman et al., 2000; Nandi and Saxena, 2020). A recently published review provides a comprehensive overview of additional molecular targets and *in-vitro* methods (Zhang et al., 2022). However, greater emphasis should be placed on validating *in-vitro* results *in-vivo*. In this context, continued *Drosophila* experiments could be implemented. Studies on glucose transport inhibition in this *in-vivo* model would be of great interest, since the first *Drosophila* glucose transporter was recently discovered in the midgut (Li Y. et al., 2021).

In summary, we suggest that the comprehensive screening toolbox proposed here could facilitate the identification of new plant-derived bioactive compounds with glucose homeostasis modulating properties.

## Abbreviation

Akt, protein kinase B; Dilp, *Drosophila* insulin-like peptides; *D. melanogaster*, *Drosophila melanogaster*; DPP4, dipeptidyl peptidase 4; GI, gastrointestinal; GIP, glucose-dependent insulinotropic polypeptide; GLP1, glucagon-like peptide-1; GLUT, glucose transporter; HET-CAM, hen’s egg test - chorioallantoic membrane; HFD, high-fat diet; HSD, high-sugar diet; IGT, impaired glucose tolerance; I_SC_, short-circuit current; PECKISH, Plant Extract Collection Kiel in Schleswig-Holstein; PI3K, phosphoinositide 3-kinase; PTP1B, protein tyrosine phosphatase 1B; SGLT1, sodium-dependent glucose cotransporter; TAG, triacylglyceride; TIRF, total internal reflection fluorescence.
